# Recent Discovery of Nitrogen Heterocycles from Marine-Derived *Aspergillus* Species

**DOI:** 10.3390/md22070321

**Published:** 2024-07-18

**Authors:** Jueying Shi, Miao Yu, Weikang Chen, Shiji Chen, Yikang Qiu, Zhenyang Xu, Yi Wang, Guolei Huang, Caijuan Zheng

**Affiliations:** 1 Key Laboratory of Tropical Medicinal Resource Chemistry of Ministry of Education, College of Chemistry and Chemical Engineering, Hainan Normal University, Haikou 571158, China; jueying202406@163.com (J.S.); yumiaonpc@126.com (M.Y.); 18971192012@126.com (W.C.); chenshijinpc@126.com (S.C.); qyk7747226@sina.com (Y.Q.); wilyq1203@163.com (Z.X.); wy718029@163.com (Y.W.); 2 Key Laboratory of Tropical Medicinal Plant Chemistry of Hainan Province, Haikou 571158, China

**Keywords:** *Aspergillus* sp., marine, secondary metabolite, nitrogen heterocycles, biological activity

## Abstract

Nitrogen heterocycles have drawn considerable attention because of their structurally novel and significant biological activities. Marine-derived fungi, especially the *Aspergillus* species, possess unique metabolic pathways to produce secondary metabolites with novel structures and potent biological activities. This review prioritizes the structural diversity and biological activities of nitrogen heterocycles that are produced by marine-derived *Aspergillus* species from January 2019 to January 2024, and their relevant biological activities. A total of 306 new nitrogen heterocycles, including seven major categories—indole alkaloids, diketopiperazine alkaloids, quinazoline alkaloids, isoquinoline alkaloids pyrrolidine alkaloids, cyclopeptide alkaloids, and other heterocyclic alkaloids—are presented in this review. Among these nitrogen heterocycles, 52 compounds had novel skeleton structures. Remarkably, 103 compounds showed various biological activities, such as cytotoxic, antimicrobial, anti-inflammatory, antifungal, anti-virus, and enzyme-inhibitory activities, and 21 compounds showed potent activities. This paper will guide further investigations into the structural diversity and biological activities of nitrogen heterocycles derived from the *Aspergillus* species and their potential contributions to the future development of new natural drug products in the medicinal and agricultural fields.

## 1. Introduction

The marine ecosystem has attracted the attention of natural product chemists due to the prospects of biologically active marine natural products. Marine natural products have attracted much attention from both natural product chemists and pharmacologists due to their novel structure and potential bioactivities [1,2,3,4]. One of the most interesting classes of marine natural products is nitrogen heterocycles. Marine-derived nitrogen-containing secondary metabolites exhibit a variety of biological activities, such as cytotoxic, antifungal, antibacterial, anti-inflammatory, and enzyme-inhibitory activities [1,5,6].

Marine-derived fungi, especially marine-derived *Aspergillus* species, are the richest sources of these basic nitrogen-containing secondary metabolites, which can produce a large number of structurally unique heterocyclic alkaloids [5,6,7]. In the present review, we have updated the diversity and biological activities of nitrogen heterocycles that are produced by marine-derived *Aspergillus* species. Herein, a total of 306 new compounds (including 13 pairs of racemates) reported from the beginning of January 2019 to January 2024 are included, and 115 references are cited in this review. We only report the previously undescribed compounds isolated during this period, while the compounds reported before this period and mentioned in the references used in this review are not included. The relevant biological and pharmacological activities of some potential alkaloids are also highlighted, which will benefit future drug development and innovation.

## 2. Structural and Biological Activity Studies

**Figure 1:**
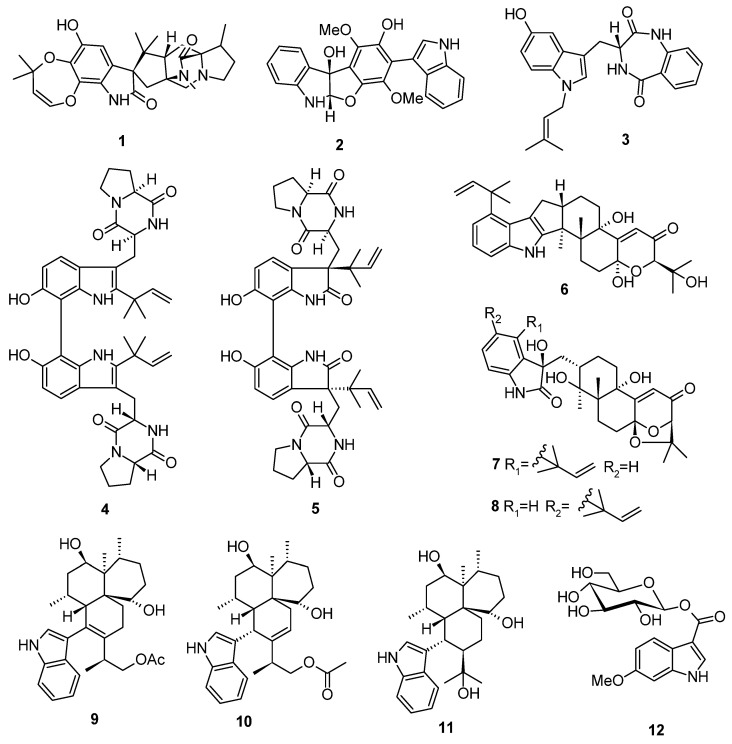

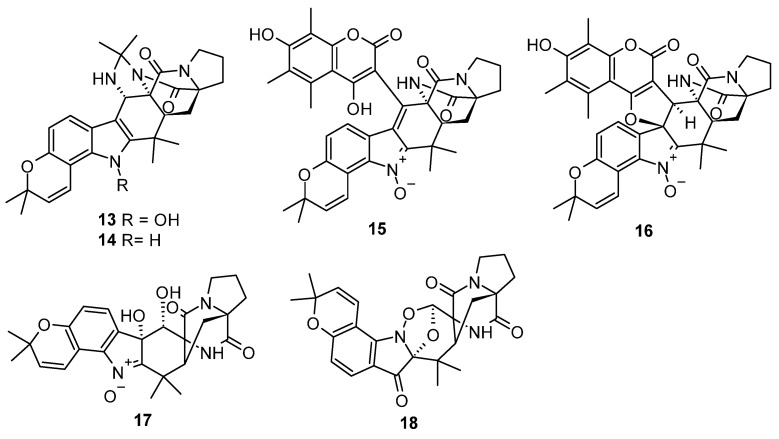
Indole alkaloids produced by marine-derived *Aspergillus* species (**1**–**18**).

**Figure 2:**
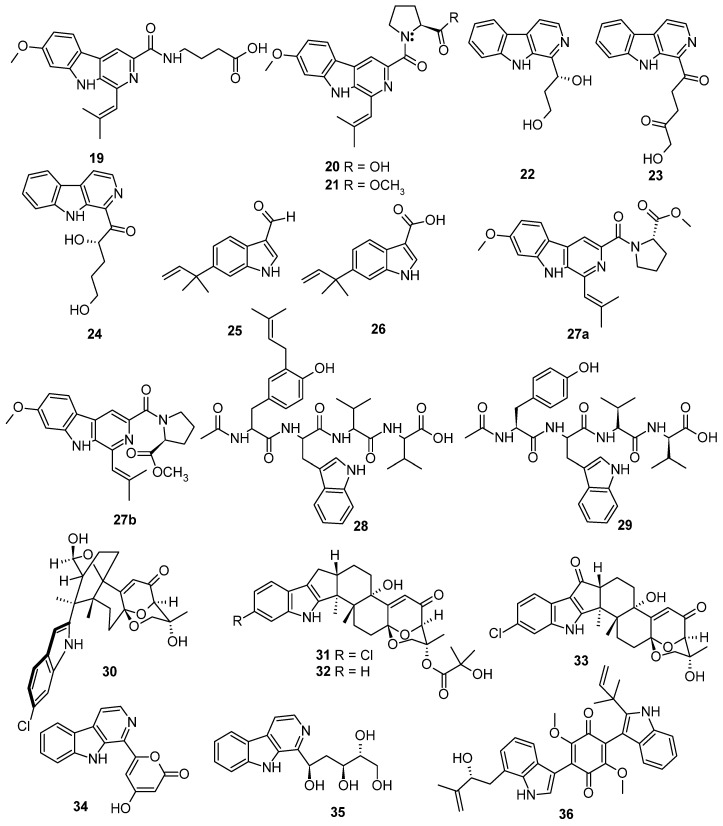

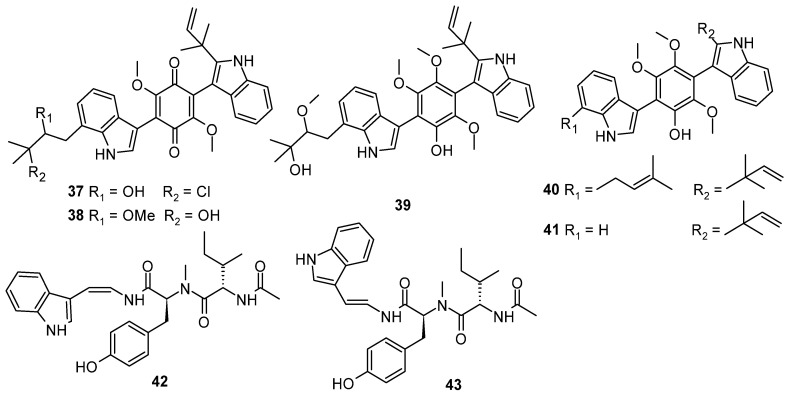
Indole alkaloids produced by marine-derived *Aspergillus* species (**19**–**43**).

**Figure 3:**
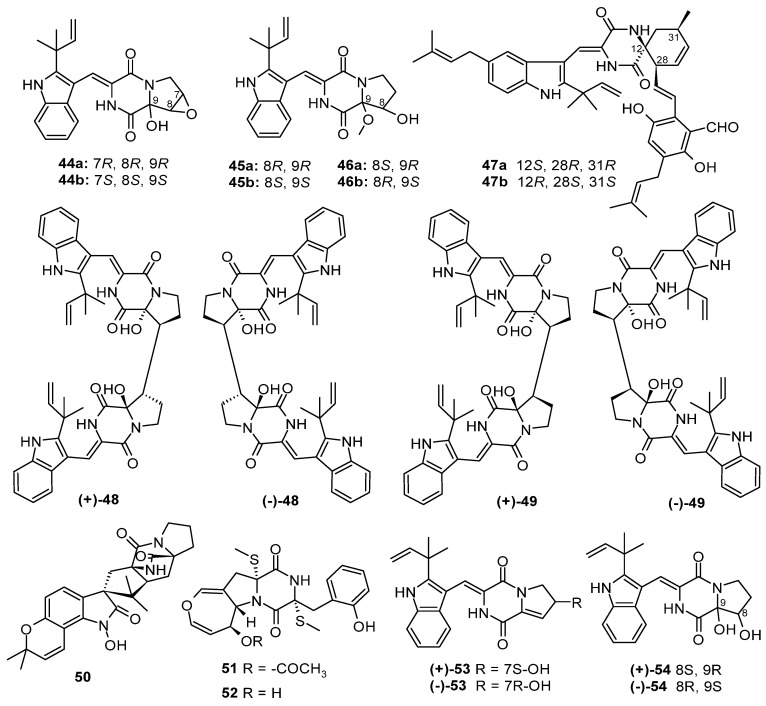
Diketopiperazine alkaloids produced by marine-derived *Aspergillus* species (**44**–**54**).

**Figure 4:**
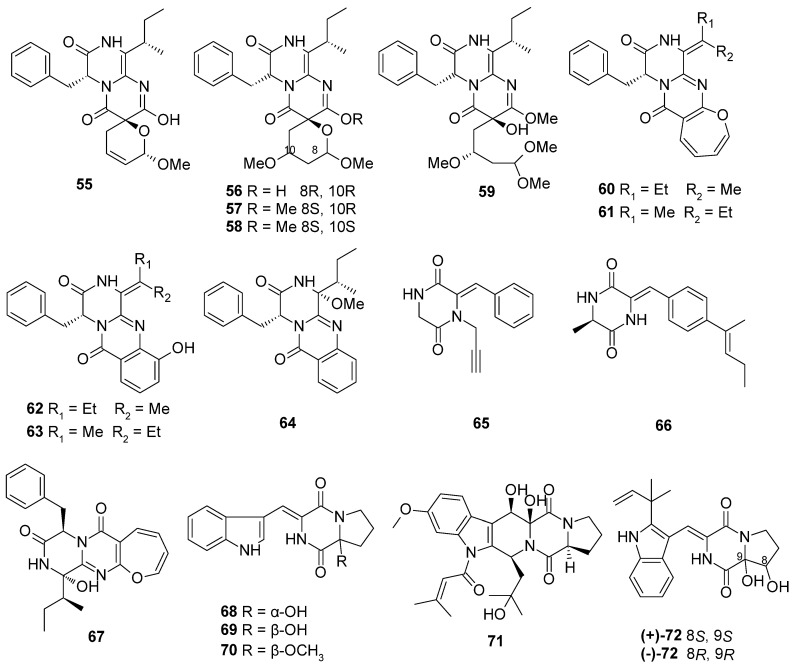
Diketopiperazine alkaloids produced by marine-derived *Aspergillus* species (**55**–**72**).

**Figure 5:**
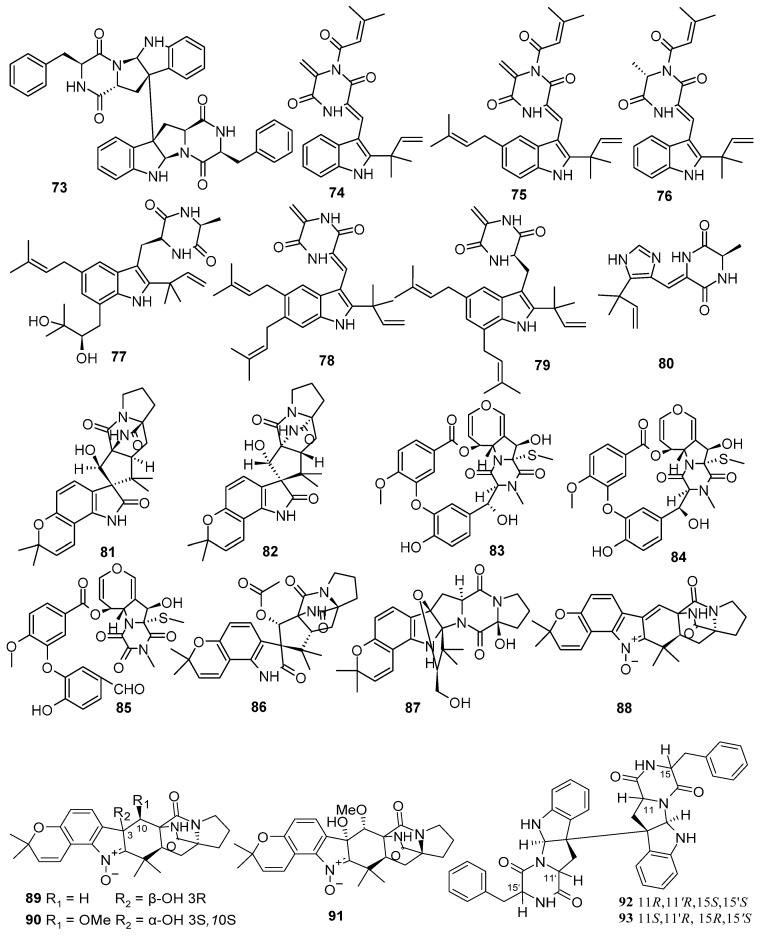
Diketopiperazine alkaloids produced by marine-derived *Aspergillus* species (**73**–**93**).

**Figure 6:**
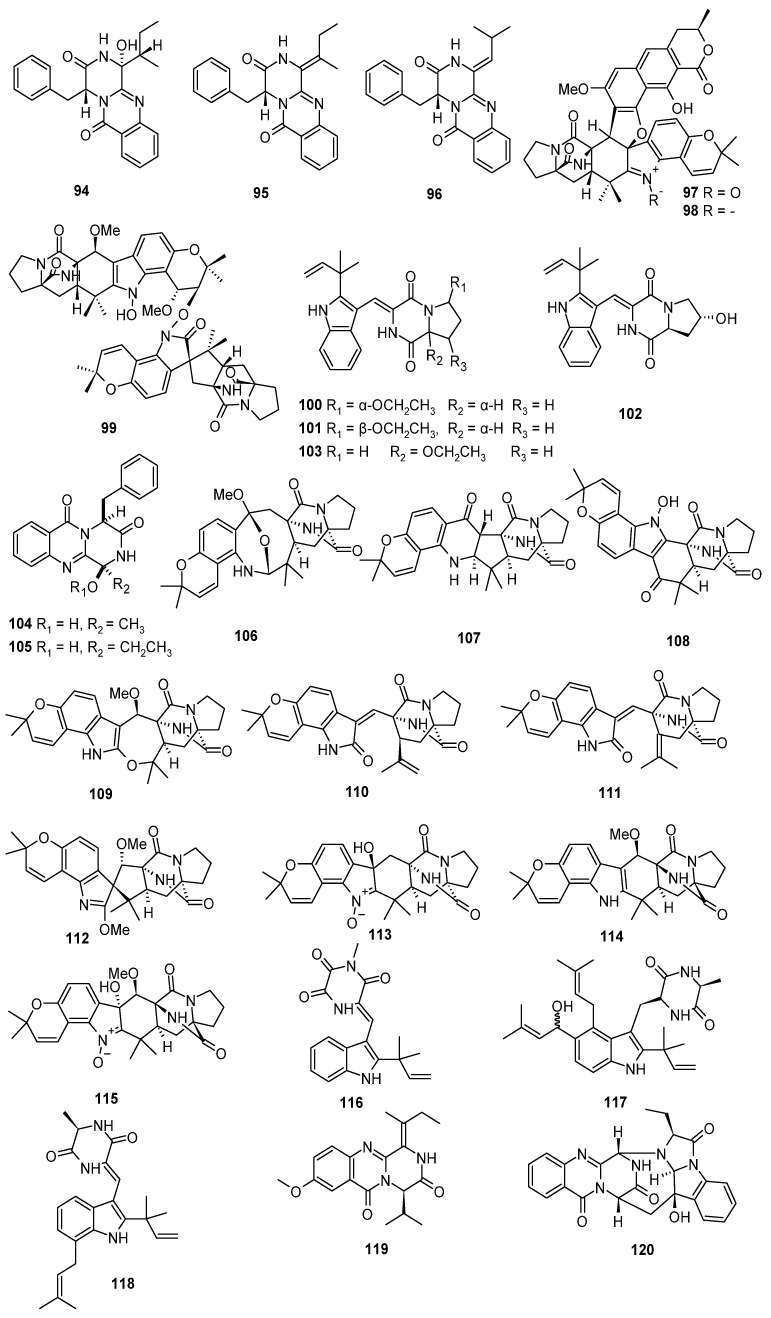
Diketopiperazine alkaloids produced by marine-derived *Aspergillus* species (**94**–**120**).

**Figure 7:**
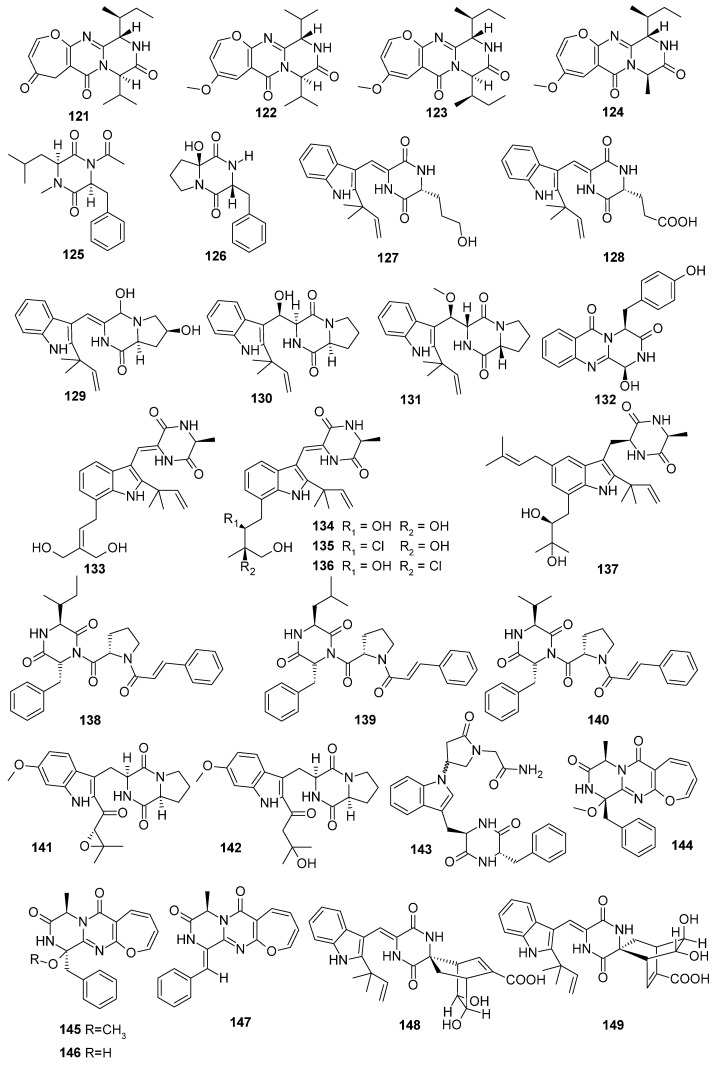
Diketopiperazine alkaloids produced by marine-derived *Aspergillus* species (**121**–**149**).

**Figure 8:**
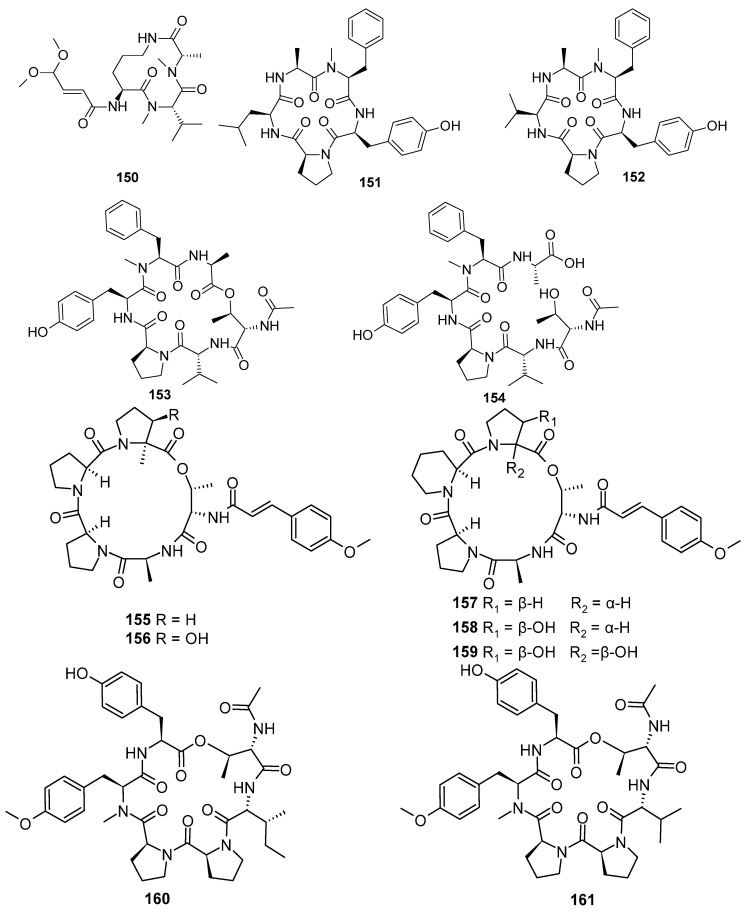
Cyclopeptide alkaloids produced by marine-derived *Aspergillus* species (**150**–**161**).

**Figure 9:**
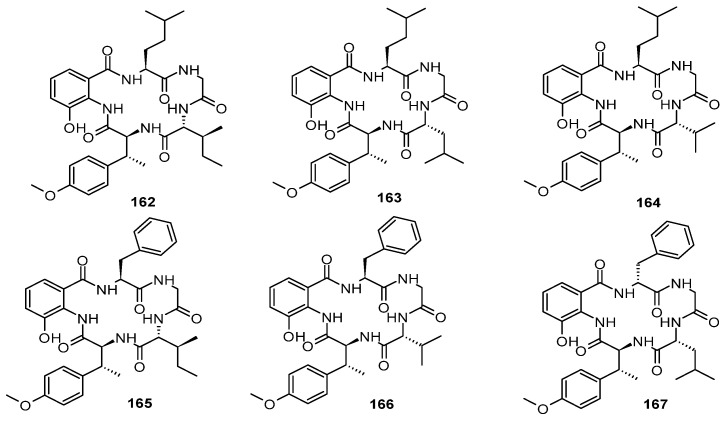

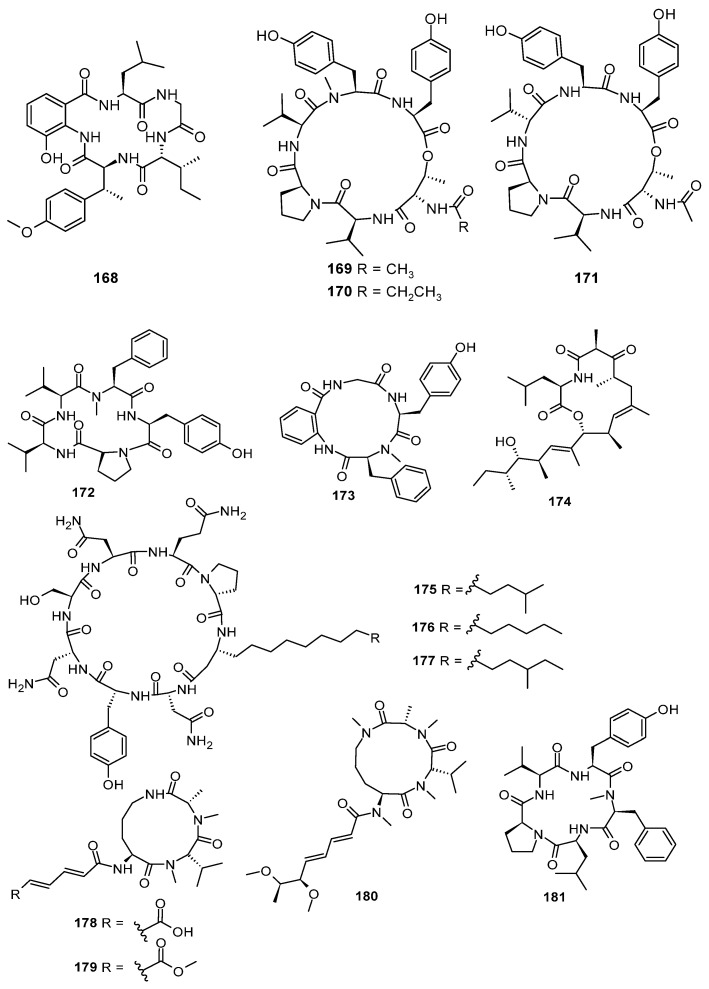
Cyclopeptide alkaloids produced by marine-derived *Aspergillus* species (**172**–**181**).

**Figure 10:**
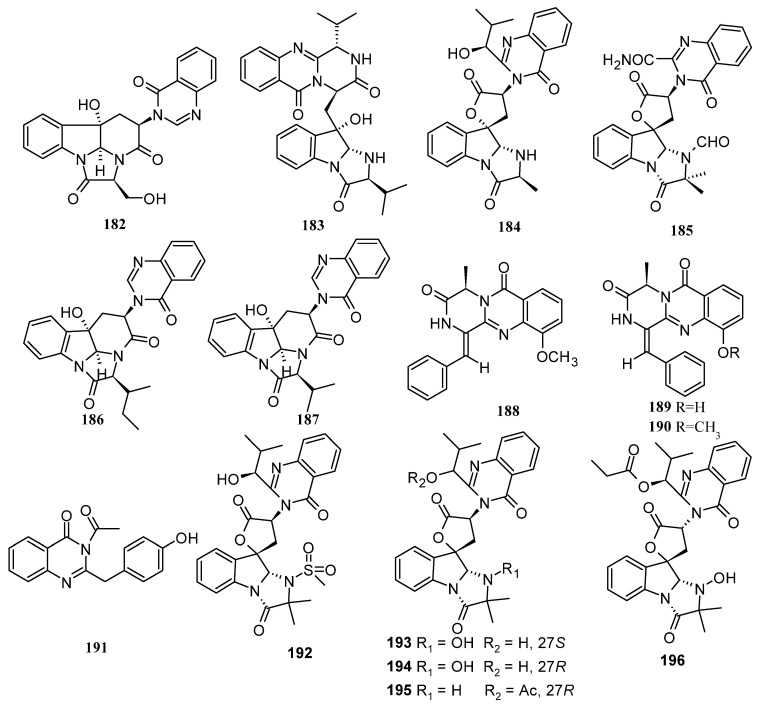

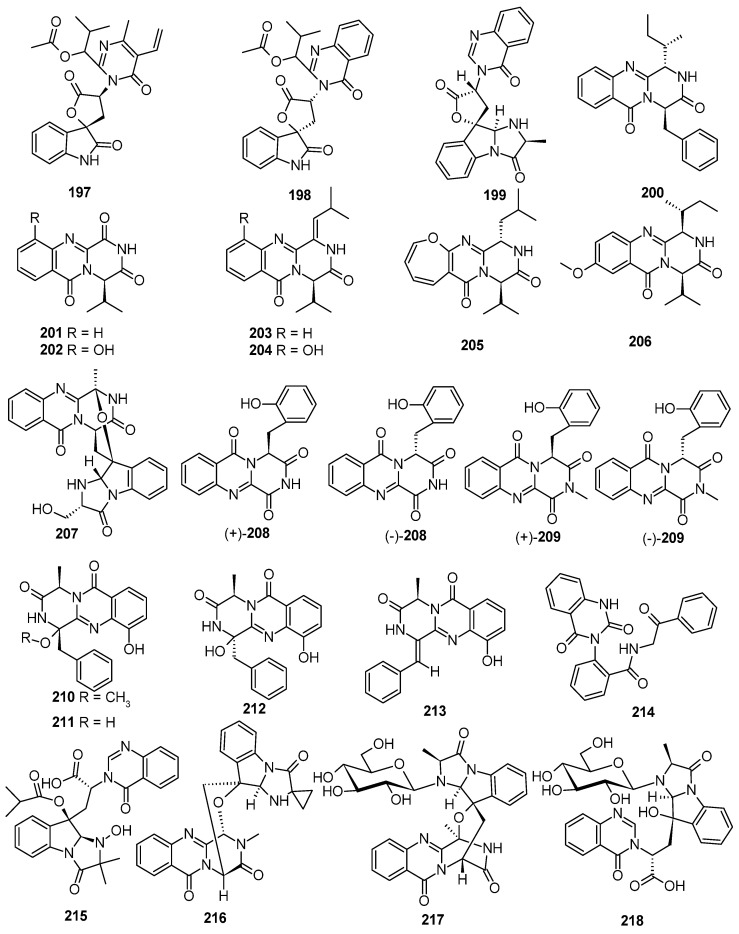
Quinazoline alkaloids produced by marine-derived *Aspergillus* species (**182**–**218**).

**Figure 11:**
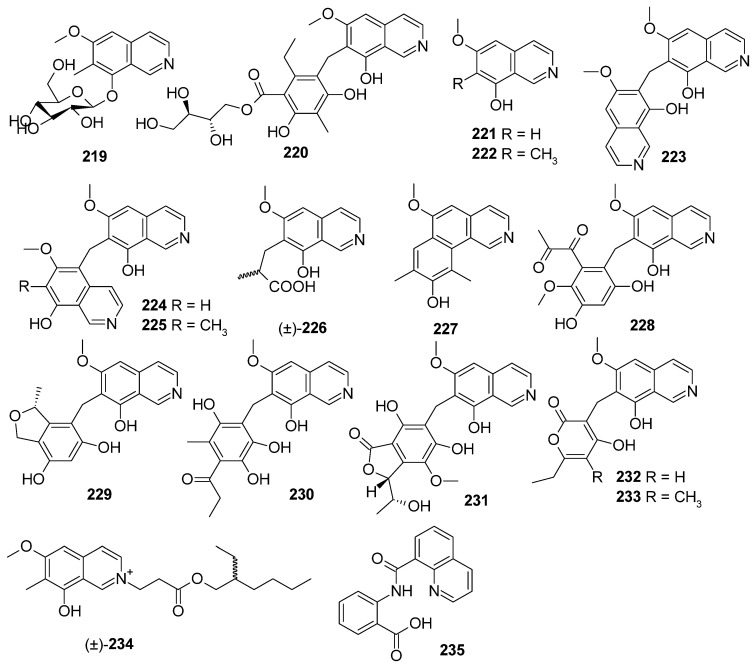
Isoquinoline alkaloids produced by marine-derived *Aspergillus* species (**219**–**235**).

**Figure 12:**
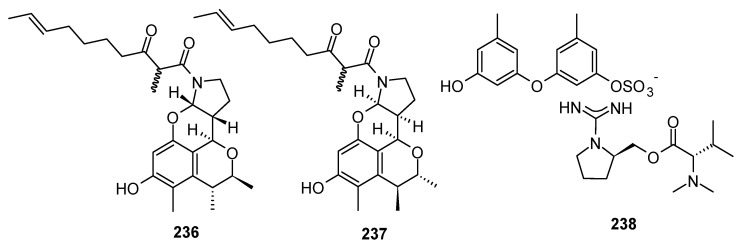

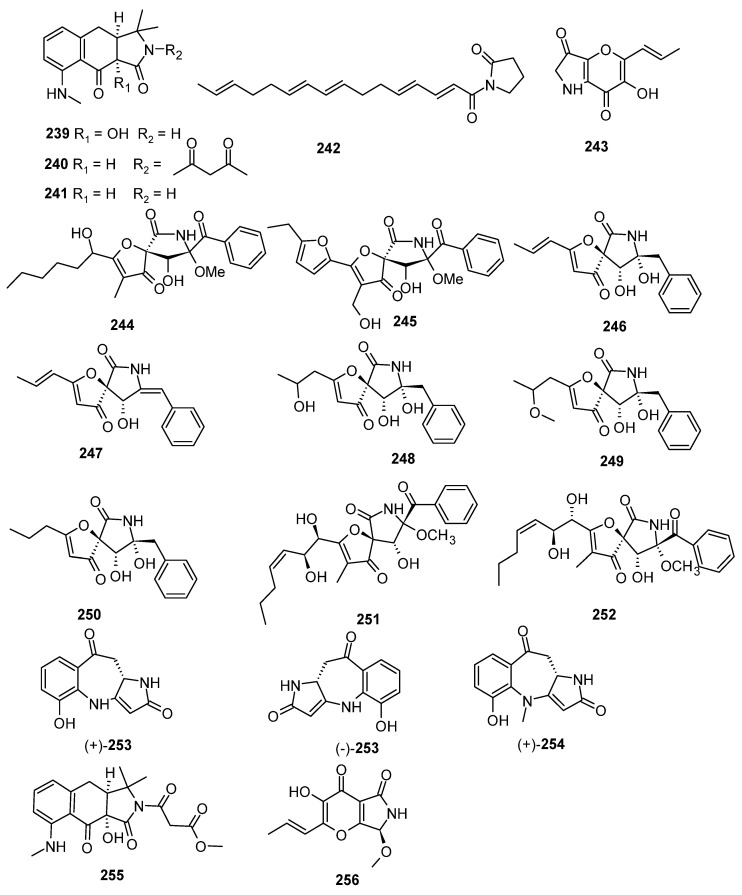
Pyrrolidine alkaloids produced by marine-derived *Aspergillus* species (**236**–**256**).

**Figure 13:**
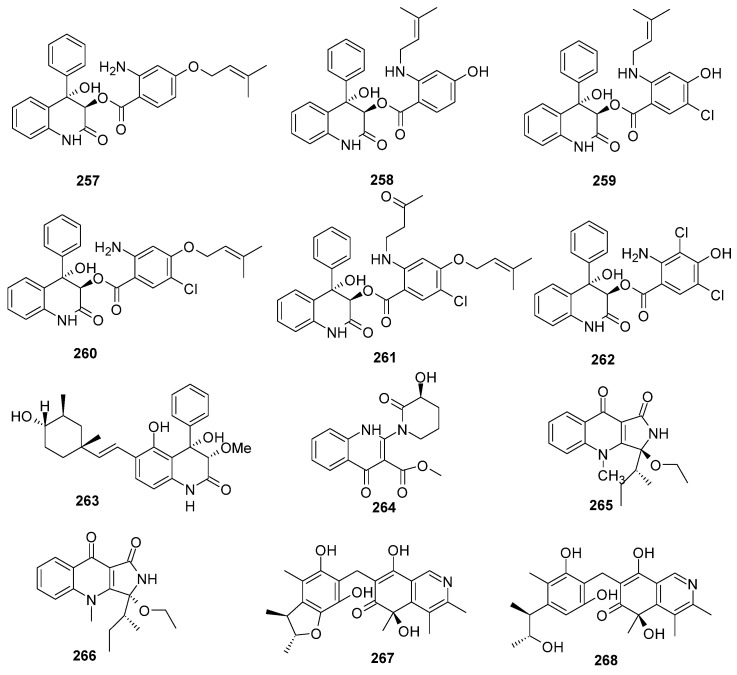
Other heterocyclic alkaloids produced by marine-derived *Aspergillus* species (**257**–**268**).

**Figure 14:**
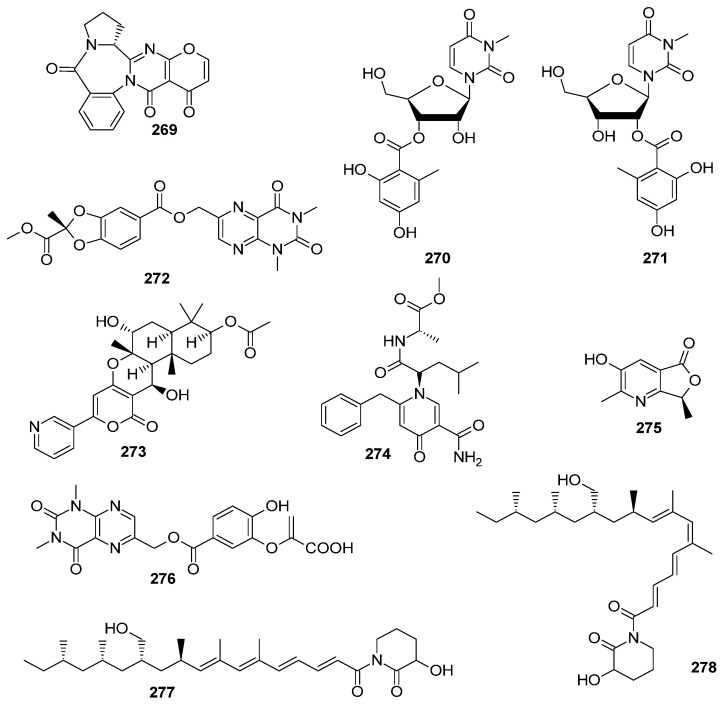
Other heterocyclic alkaloids produced by marine-derived *Aspergillus* species (**269**–**278**).

**Figure 15:**
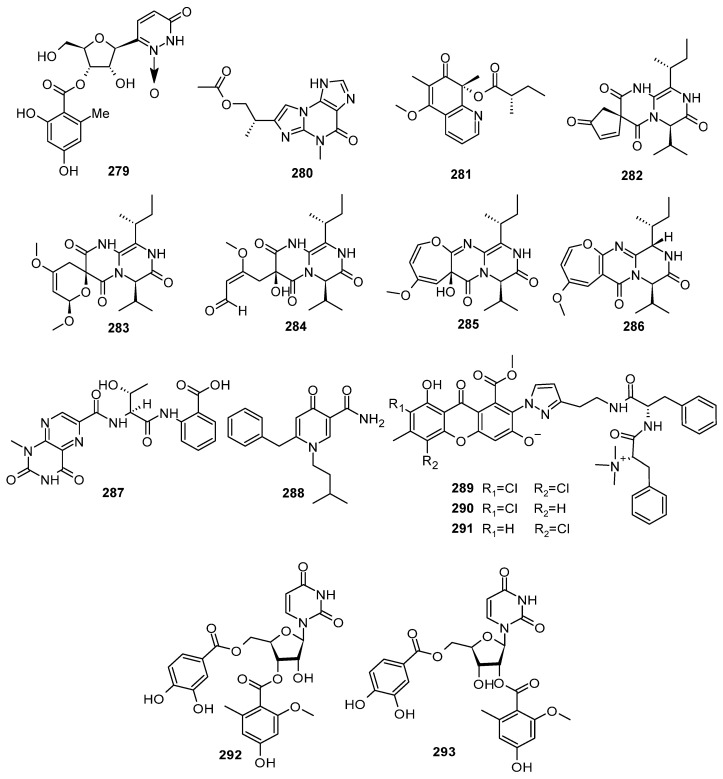
Other heterocyclic alkaloids produced by marine-derived *Aspergillus* species (**279**–**293**).

Based on the literature search, 306 previously undescribed nitrogen heterocycles (**1**–**293**, including 13 pairs of racemates) were obtained from marine-derived *Aspergillus* species from 2019 to 2024 (Figure 1, Figure 2, Figure 3, Figure 4, Figure 5, Figure 6, Figure 7, Figure 8, Figure 9, Figure 10, Figure 11, Figure 12, Figure 13, Figure 14 and Figure 15). These heterocyclic alkaloids could be classified into seven major categories—indole alkaloids (**1**–**43**), diketopiperazine alkaloids (**44**–**149**), cyclopeptide alkaloids (**150**–**181**), quinazoline alkaloids (**182**–**218**), isoquinoline alkaloids (**219**–**235**), pyrrolidine alkaloids (**236**–**256**), and other heterocyclic alkaloids (**257**–**293**)—based on their structural patterns. The racemates were purified by chiral HPLC analysis. The structures and the absolute configurations of the new compounds and novel skeleton compounds were elucidated by a detailed spectroscopic analysis of NMR and MS data, time-dependent density functional theory (TDDFT)/ECD calculations, DP4+ probability predictions, as well as single-crystal X-ray diffraction. The absolute configurations of the amino acid residues of the peptides were determined by Marfey’s method.

### 2.1. Indole Alkaloids

Indole alkaloids are a class of alkaloids containing an indole moiety. The indole moiety, presented in a wide range of marine natural products, showed various bioactivities, which may be one of the most important components of the heterocycles in the discovery of new drugs [7,8,9]. A total of 44 new indole alkaloids (**1**–**43**, including 1 pair of racemates) were discovered from marine-derived *Aspergillus* species, including 11 compounds with novel skeleton structures (Figure 1 and Figure 2). Remarkably, 14 of them showed cytotoxic, pro-angiogenic, protein tyrosine phosphatase 1B-inhibitory, antibacterial, antifungal, and antivirus activities. Intriguingly, many of these indole alkaloids possess unique structural features as well as interesting biological and pharmacological activities.

One new paraherquamide, aculeaquamide A (**1**), was isolated from the marine-derived fungus *Aspergillus aculeatinus* WHF0198. Compound **1** showed cytotoxicity against Bel-7402 with an IC_50_ value of 3.3 μM [10]. A new indole alkaloid, asterriquinone F (**2**), was isolated from the brown alga-derived fungus *Aspergillus terreus* LM.1.5 [11]. A new tryptoquivaline analog, asperdiazapinone G (**3**), was isolated from the mangrove soil-derived fungus *Aspergillus* sp. WHUF03110 [12]. Two new prenylated indole alkaloid homodimers, di-6-hydroxydeoxybrevianamide E (**4**) and dinotoamide J (**5**), were obtained from the seawater-derived fungus *Aspergillus austroafricanus* Y32-2. Compound **5** exhibited pro-angiogenic activity in a PTK787-induced vascular injury zebrafish model at the concentration of 70 µg/mL [13]. Six new indole-diterpenoids, penerpene O (**6**), penerpenes Q–R (**7**–**8**) and penerpenes T–V (**9**–**11**), were isolated from the marine soft coral-derived fungus *Aspergillus* sp. ZF-104. Compound **8** bears a rare indolin-2-one unit in its structure, and **9** contains a reconstructed novel skeleton in which the indole ring and the terpenoid substructure were cleaved before being reconnected through the nitrogen atom. Compounds **6** and **10** showed protein tyrosine phosphatase 1B (PTP1B)-inhibitory activities, with IC_50_ values of 17.7 ± 0.7 and 28.1 ± 2.2 μM, respectively, comparable to that of the positive control NaVO_3_ (IC_50_, 33.6 ± 0.92 μM) [14]. One new indole glucoside, 6-methoxyindole-3-carboxylic acid O-*β*-*D*-glucopyranosyl ester (**12**), was isolated from sea cucumber-derived fungus *Aspergillus fumigatus* M580 [15]. Six new alkaloids, sclerotiamides C−H (**13**–**18**), were isolated from gorgonian-derived fungus *Aspergillus sclerotiorum* LZDX-33-4. Compounds **13** and **14** were notoamide-type alkaloids with the incorporation of a unique 2,2-diaminopropane unit, **15** and **16** were unprecedented notoamide hybrids with a new coumarin unit. Compound **18** represented a new highly oxidized notoamide scaffold. The whole genome was sequenced and analyzed using antiSMASH, in order to deduce the biogenetic pathway of the unprecedented notoamides. Compound **13** showed significant inhibitory activity against HeLa, A549, HepG2, and SMMC7721 cell lines with IC_50_ values of 1.7 ± 0.1, 1.6 ± 0.1, 1.8 ± 0.1, and 1.5 ± 0.1 μM, respectively. Compound **16** showed significant inhibitory activity against HeLa, A549, HepG2, and SMMC7721 cell lines with IC_50_ values of 7.9± 0.2, 7.8 ± 0.1, 8.1 ± 0.2, and 6.7 ± 0.2 μM, respectively. The preliminary study of mechanism indicated that **13** induced apoptosis in HeLa cells by arresting the cell cycle, activating ROS production, and regulating apoptosis-related proteins in the MAPK signaling pathway. The significant anti-HeLa effect of **13** suggested it to be a potential lead compound for further development as an anti-cervical tumor agent [16] (Figure 1).

Three new indole alkaloids, fumindolines, A–C (**19**–**21**), were obtained from the marine seawater-derived fungus *Aspergillus fumigatus* H22 [17]. Three new *β-*carboline alkaloids, aspercarbolines A–C (**22**–**24**), were isolated from the fungus *Aspergillus* sp. XBB-4 was isolated from the inner tissue of geoduck *Panopea abbreviate*, which was collected from the South China Sea. Compound **24** exhibited significant cytotoxic activity against human nasopharyngeal carcinoma cell lines (CNE1, CNE2, HONE1, and SUNE1) and human hepatocellular carcinomacell lines (hepG2 and QGY7701), with IC_50_ values of 16.29, 20.58, 20.11, 45.31, 50.85, and 28.97 μM, respectively [18]. Two new indole alkaloids, flavonoids A (**25**) and B (**26**), were isolated from the marine deep-sea-derived fungus *Aspergillus flavipes* DS720. Compound **25** showed high and broad-spectrum cytotoxic activities against HeLa, 5637, CAL-62, PATU8988T, A-375, and A-673 cell lines, with inhibition rates of (96.94 ± 0.62)%, (99.49 ± 0.50)%, (96.16 ± 1.34)%, (90.83 ± 3.31)%, (99.32 ± 0.11)%, and (90.01 ± 5.81)%, respectively, at the concentration of 20 µM, indicating that **25** may possess certain potential for the development of lead compounds in the future [19]. A pair of inseparable mixtures of secofumitremorgins A (**27a**) and B (**27b**), which differed in the configuration of the nitrogen atom, were isolated and identified from the deep-sea sediment-derived fungus *Aspergillus fumigatus* SD-406. Their structures were determined through a detailed spectroscopic analysis of NMR and MS data, a chiral HPLC analysis of the acidic hydrolysate, an X-ray crystallographic analysis, a *J*-based configuration analysis, and quantum chemical calculations of ECD, OR, and NMR (with DP4+ probability analysis). Compounds **27**, **27a,** and **27b** exhibited activity against aquatic pathogenic bacteria *Vibrio alginolyticus* and *Edwardsiella tarda* and the plant pathogenic fungi *Fusarium graminearum* Schw, with minimum inhibitory concentration (MIC) values of 32, 64, and 4 µg/mL, respectively [20]. Aspergillipeptides H–I (**28**–**29**) were isolated from the marine gorgonian-associated fungus *Aspergillus* sp. SCSIO 41501 [21]. Four new indole diterpenoids, ascandinines A–D (**30**–**33**), were isolated from an Antarctic sponge-derived fungus *Aspergillus candidus* HDN15-152. Compound **30** possesses an unprecedented 2-oxabicyclo [2.2.2]octan-3-ol motif embedded in a pentacyclic ring system, while **31–33** represent a rare type of indole diterpenoid featuring the 6/5/5/6/6/6/6-fused ring system. Compound **32** displayed anti-influenza virus A (H1N1) activity with an IC_50_ value of 26 μM, while compound **33** showed cytotoxicity against HL-60 cells with an IC_50_ value of 7.8 μM [22]. Two new *β*-carboline alkaloids, aspergillspins A–B (**34**–**35**), were isolated from the marine gorgonian-derived fungus *Aspergillus* sp. SCSIO 41501 [23]. Three new bisindolylquinones, asterriquinones I–K (**36**–**38**), and three new bis-indolylbenzenoids asterriquinols G–I (**39**–**41**), were isolated from the sponge-derived fungus *Aspergillus* sp. SCSIO 41018. Asterriquinone I (**41**) represented the first reported bis-indolylquinone possessing a chlorine atom. Compound **36** exhibited cytotoxic activities against K562, BEL-7042, and SGC-7901 cell lines, with IC_50_ values of 17.9 ± 0.62, 25.8 ± 0.16, and 29.2 ± 0.32, respectively. Compound **37** displayed cytotoxic activities against K562, BEL-7042, and SGC-7901 cell lines, were IC_50_ values of 8.5 ± 0.17, 11.1 ± 0.22, and 18.7 ±0.45 μM, respectively. Compound **38** showed cytotoxic activities against K562, BEL-7042, and SGC-7901 cell lines, with IC_50_ values of 13.0 ± 0.36, 14.9 ± 0.55, and 26.2 ± 0.13 μM, respectively [24]. Two new isomeric modified tripeptides, aspergillamides C and D (**42** and **43**), were obtained from the marine sponge *Callyspongia* sp.-derived fungus *Aspergillus terreus* SCSIO 41008 [25] (Figure 2).

### 2.2. Diketopiperazine Alkaloids

Diketopiperazine alkaloids have proven to be the most abundant heterocyclic alkaloids up to now, usually possessing diverse scaffolds and rich biological activities. Diketopiperazine alkaloids are widely distributed in filamentous fungi, especially in the genera *Aspergillus* and *Penicillium* of the phylum Ascomycota or sac fungi [26,27]. A total of 115 new diketopiperazine alkaloids (**44**–**149**, including 9 pairs of racemates) were discovered from marine-derived *Aspergillus* species (Figure 3, Figure 4, Figure 5, Figure 6 and Figure 7), 18 of them with novel skeleton structures. Thirty-seven of them showed anti-virus, cytotoxic, inhibit thioredoxin reductase, antibacterial, antifungal, anti-inflammatory, *α*-glucosidase-inhibitory, and angiotensin-converting enzyme-inhibitory activities.

Six new diketopiperazines, (±)-7,8-epoxy-brevianamide Q ((±)−**44**), (±)-8-hydroxy-brevianamide R ((±)−**45**), and (±)-8-epihydroxy-brevianamide R ((±)−**46**), were isolated from a marine sediment-derived fungus *Aspergillus versicolor* MF180151. Compound **44** is the first sample of brevianamides with an epoxy moiety. The racemates of **44**–**46** were isolated by chiral HPLC analysis, and their structures were clarified using the calculated ECD, and DP4+ probability methods. [28]. A pair of new spirocyclic alkaloids, (-)-5-isopentenyl-cryptoechinuline D (**47a**) and (+)-5-isopentenyl-cryptoechinuline D (**47b**), were obtained from a marine moss-derived fungus *Aspergillus ruber* TX-M4-1. Compound **47a** was shown to inhibit thioredoxin reductase (TrxR) activity with an IC_50_ value of 6.2 μmol/L [29]. Two pairs of new dimeric diketopiperazine alkaloids, (±)-dibrevianamides Q1 ((±)-**48**) and Q2 ((±)-**49**), were obtained from a marine-derived fungus *Aspergillus* sp. ZA-01. Compounds (+)-**48** and (−)-**49** were clarified using the calculated ECD and DP4+ probability methods. Compounds **48** and **49** displayed anti-H1N1 virus activity, with IC_50_ values of 12.6 and 19.5 μM, respectively. Compound (+)-**48** showed significant activity against *Mycobacterium tuberculosis* (MIC, 10.2 μg/mL) [30]. A new diketopiperazine alkaloid, versicolamide C (**50**), was isolated from a soft coral-derived fungus *Aspergillus* sp. SCSIO 41036 [31]. Two new thiodiketopiperazines, emestrins L (**51**) and M (**52**), were obtained from the sea hare *Aplysia pulmonica*-derived fungus *Aspergillus terreus* RA2905 [32]. Two pairs of diketopiperazine alkaloids, (±)-brevianamide Z [(±)-**53**] and (±)-brevianamide Z1 [(±)-**54**], were isolated from the sea mud-derived fungus *Aspergillus versicolor.* Their absolute configurations were assigned based on extensive spectroscopic analysis and calculated using the NMR, ECD, ORD, and DP4+ methods [33] (Figure 3).

Ten new diketopiperazine alkaloids, pyranamides A–D (**55**–**58**), secopyranamide C (**59**), and protuboxepins F–J (**60**–**64**), were isolated from the marine sponge-derived fungus *Aspergillus versicolor* SCSIO 41016. Compounds **55**–**58** were obtained as rare 1-oxa-8,10-diazaspiro [5.5]undecane containing diketopiperazine alkaloids, while **59** was obtained as a tetrahydropyran-ring cleavage derivative. Plausible biosynthetic pathways of **55**–**64** were postulated [34]. Two new diketopiperazine alkaloids, sclerotioloids A (**65**) and C (**66**), were obtained from the sponge-derived fungus *Aspergillus sclerotiorum* ST0501 [35]. One new oxepin-containing diketopiperazinetype alkaloid, oxepinamide L (**67**), was isolated from the culture of the marine coral-derived fungus *Aspergillus puniceus* [36]. Three new diketopiperazine alkaloids, asperindopiperazines A–C (**68**–**70**), were isolated from the Mariana-Trench-associated fungus *Aspergillus* sp. SY2601 [37]. One new diketopiperazine alkaloid, 12*β*,13*β*-hydroxy-asperfumigatin (**71**), was isolated from the marine-derived fungus *Aspergillus fumigatus* H22 [17]. Two new diketopiperazine alkaloids, (+)- and (−)-brevianamide X ((±)-**72**), were isolated from the seaweed *Enteromorpha prolifera*-derived fungus *Aspergillus versicolor* OUCMDZ-2738. Their structures were elucidated based on spectroscopic analysis, specific rotation analysis, ECD, and X-ray crystallographic analysis. Compound **72** was further resolved into the corresponding optically pure enantiomers and its absolute configurations were determined for the first time. A plausible biosynthetic relationship of **72**, through a sequence of oxidative transformations and hydrolysis or methanolysis, was illustrated [38] (Figure 4).

A new asymmetric diketopiperazine dimer, asperflocin (**73**), was isolated from the sponge-associated fungus *Aspergillus versicolor* 16F-11. Compound **73** showed moderate cytotoxic activity against A375 cell lines, with an IC_50_ value of 10.29 ± 2.37 μM [39]. Four new indole diketopiperazine alkaloids, aspechinulins A-D (**74**–**77**), were isolated from the deep-sea sediment-derived fungus *Aspergillus* sp. FS445. Compounds **74**–**76** represented the first examples of indole diketopiperazine derivatives constructing a C_5_ unit at 11-NH through an imide linkage. Compound **76** exhibited potential inhibitory activities against NO production with the same IC_50_ value of the positive control aminoguanidine (IC_50_, 23.7 μM) [40]. Two new diketopiperazines, 5-prenylcryptoechinulin A (**78**) and 9-*epi*-didehydroechinulin (**79**), were obtained from the deep-sea-derived fungus *Aspergillus chevalieri* MCCC M23426. Compound **78** displayed selective antibacterial activities against *Staphylococcus aureus* CICC 10384, with an inhibition rate of over 90%, at the concentration of 250 µM [41]. One new piperazinedione, asperdione A (**80**), was isolated from the *Aspergillus* sp. XBB-4, which was isolated from the inner tissue of geoduck *Panopea abbreviate,* which was collected from the South China Sea. Compound **80** exhibited significant cytotoxic activity against human nasopharyngeal carcinoma cell lines (CNE1, CNE2, HONE1 and SUNE1) and human hepatocellular carcinomacell lines (hepG2 and QGY7701), with IC_50_ values of 22.00, 18.93, 21.61, 16.93, 12.33, and 10.72, respectively [18]. Two new diketopiperazine alkaloids, (+)19-*epi*-sclerotiamide (**81**) and (–)19-*epi*-sclerotiamide (**82**), were isolated from a soft coral-associated epiphytic fungus *Aspergillus versicolor* CGF 9-1-2 [42]. Three new emestrin-type thiodiketopiperazines, didethio-11*α*-methylthioemestrin (**83**), 7′-*epi*-didethio-11*α*-methylthioemestrin (**84**), and 2″-desmethyl-MPC1001F (**85**), were isolated and identified from the deep-sea cold seep sediment-derived fungus *Aspergillus nidulans* SD-531. Compound **85** exhibited significant antimicrobial activity against *E. tarda*, *V. alginolyticus*, *P. aeruginosa,* and *V. parahaemolyticus*, with MIC values of 0.5, 1.0, 16.0, and 16.0 μg/mL, respectively. Compound **85** showed cytotoxic activity against the tumor cell line Huh 7.5, with an IC_50_ value of 8.0 μΜ [43]. Six new prenylated indole diketopiperazine alkaloids, asperthrins A–D (**88**–**91**), asperthrin E (**86**), and asperthrin F (**87**), were isolated from the marine-derived fungus *Aspergillus* sp. YJ191021. Compound **88** exhibited moderate antifungal and antibacterial activities against *V. anguillarum*, *Xanthomonas oryzae* pv. *Oryzicola*, and *Rhizoctonia solani,* with MIC values of 8, 12.5, and 25 µg/mL, respectively. Furthermore, **88** displayed notable anti-inflammatory activity, with an IC_50_ value of 1.46 ± 0.21 µM, in *Propionibacterium acnes*-induced human monocyte cell line (THP-1) [44]. Two new dimeric diketopiperazines, stereoisomers (**92**–**93**), were isolated from the culture broth of the marine-derived fungus *Aspergillus* sp. Z3, which was found in the gut of marine isopod *Ligia exotica* [45] (Figure 5).

Three new diketopiperazine alkaloids, 3-hydroxyprotuboxepin K (**94**), 3,15-dehydroprotuboxepin K (**95**), and versiamide A (**96**), were isolated from the marine red algal *Rhodomela confervoides*-derived fungus *Aspergillus creber* EN-602. Compound **96** represented the first example of a naturally occurring quinazolinone alkaloid with a diketopiperazine ring derived from phenylalanine (Phe) and leucine (Leu). Compound **94** exhibited inhibitory activity against the angiotensin-converting enzyme (ACE) with an IC_50_ value of 22.4 μM. Compound **95** exhibited antimicrobial activity against *E. tarda*, *E. coli* and *M. luteus*, *P. aeruginosa,* and *V. harveyi*, with MIC values of 64, 8 and 16, 32 and 64 μg/mL, respectively. Compound **96** exhibited antimicrobial activity against *Aeromonas hydrophila*, *E. coli*, *M. luteus,* and *P. aeruginosa*, with MIC values of 64, 16, 64, and 64 μg/mL, respectively [46]. Three new diketopiperazine derivatives, Waikikiamides A–C (**97**–**99**), were isolated from a Hawaiian marine-derived fungus, *Aspergillus* sp. FM242. Compound **99** featured the first unique heterodimer of two notoamide analogs with an N-O-C bridge. The hybrids of dipeptidylpyruvate and polyketides (**97**–**98**) had quite rare secondary metabolites, and the dipeptidylpyruvate heterodimer (**99**) with an N-O-C bridge is unprecedented. The discovery of **97**–**99** expanded the chemical diversity of the known dipeptidylpyruvate scaffolds and provided intriguing templates for synthetic and biosynthetic chemists. The Waikikiamide biosynthetic gene cluster derived from the complete sequencing and bioinformatic mining of the *Aspergillus* sp. FM 242 genome. The gene clusters mined from the sequenced genome support their putative biosynthetic pathways. Compound **97** showed antiproliferative activity against cell lines (HT1080, PC3, Jurkat, A2780S), with IC_50_ values of 0.519, 1.855, 0.62, and 0.78 μM, respectively. Compound **99** exhibited antiproliferative activity against cell lines (HT1080, PC3, Jurkat, A2780S), with IC_50_ values of 1.135, 1.805, 1.79, and 1.127 μM, respectively. Furthermore, compound **97**, a dipeptidylpyruvate-polyketide hybrid with a nitrogenated methoxy group, showed the most antiproliferative activity, and could potentially attract widespread attention from cancer biologists, biochemists, medicinal chemists, and pharmacologists focusing on new anticancer drug discovery and development [47]. Four new indolyl diketopiperazines, aspamides A–D (**100**–**103**), and two new diketopiperazines, aspamides F–G (**104**–**105**), were isolated from the solid culture of *Aspergillus versicolor*, which is an endophyte with the sea crab (*Chiromantes haematocheir*). All compounds were selected for virtual screening on the 3CL hydrolase (Mpro) of SARS-CoV-2, which was exploited as a potential drug target to fight COVID-19. The docking scores of compounds **100**, **101**, **104,** and **105** were top among all screened molecules (docking scores: −5.389; −4.772; −5.146; −4.962; −5.158), and the score of ritonavir (a potent in vitro inhibitor of human immunodeficiency virus type 1 protease) was −7.039, suggesting that these compounds may be helpful in fighting COVID-19, although further studies are required [48]. Ten prenylated notoamide-type alkaloids, sclerotiamides I-R (**106**–**115**), were isolated from marine gorgonian-derived fungus *Aspergillus sclerotiorum* LZDX-33-4. Compound **107** possessed inhibitory effects against LDH and IL-1*β* expression in BV-2 cells. Further investigation revealed that **107** significantly inhibited NLRP3 inflammasome activation and blocked NLRP3 inflammasome-induced pyroptosis via the amelioration of mitochondria damage. Compound **108** exhibited potent inhibition against pathogenic *S. aureus* ATCC 29213, MRSA T144, and *E. faecalis* ATCC 29212, with MIC values of 4.0, 4.0, and 16.0 μM, respectively [49]. Three new indole diketopiperazine alkaloids, 11-methylneoechinulin E (**116**) and variecolorin M (**117**), and (+)-variecolorin G (**118**), were isolated from a soft coral-associated epiphytic fungus *Aspergillus* sp. EGF 15-0-3. The enantiomeric mixtures and (+)-variecolorin G (**118**), with a ratio of 1:2, were separated using chiral HPLC separation, and the absolute configurations of **118** were determined by experimental and quantum-chemical ECD investigations and single-crystal X-ray diffraction analysis [50]. Two new quinazolinone diketopiperazine alkaloids, versicomide E (**119**) and cottoquinazoline H (**120**), were isolated from deep-sea coral *Hemicorallium* cf. *Imperiale-*derived fungus *Aspergillus versicolor* AS-212. Compound **120** exhibited inhibitory effects against *Vibrio harveyi* and *V. parahaemolyticus* with MIC values of 18.1 and 9.0 µM, respectively [51] (Figure 6).

Four new oxepine-containing pyrazinopyrimidine alkaloids, versicoxepines A–D (**121**–**124**), were isolated from *Aspergillus versicolor* AS-212, a fungus isolated from the deep-sea coral *Hemicorallium* cf. *imperiale*. Compounds **122** and **123** represented the first example of a new oxepine-containing pyrazinopyrimidine alkaloid whose cyclic dipeptide moiety is composed of the same type of amino acid (Val or Ile) [52]. Two new dipeptides, asperopiperazines A and B (**125** and **126**), were isolated from the tunicate-derived *Aspergillus* sp. DY001. Compound **125** displayed antimicrobial activity against *E. coli* and *S. aureus* with the same MIC value of 8 μM. Compound **126** displayed antimicrobial effects against *E. coli* and *S. aureus,* with MIC values of 4 and 8 μM, respectively. Compound **125** displayed growth-inhibitory effects towards HCT 116 and MDA-MB-231 cells, with IC_50_ values of 15.1 ± 0.1 and 24.3 ± 0.2 μM, respectively. Compound **126** displayed growth-inhibitory effects towards HCT 116 and MDA-MB-231 cells, with IC_50_ values of 16.2 ± 0.1 and 26.3 ± 0.3 μM, respectively [53]. Six new diketopiperazine alkaloids, aspergiamides A–F (**127**–**132**), were isolated from the mangrove *Sonneratia apetala* endophytic fungus *Aspergillus* sp. 16-5c. Compound **127** exhibited significant *α*-glucosidase-inhibitory effects, with an IC_50_ value of 18.2 µM. Compound **129** exhibited moderate *α*-glucosidase inhibition with an IC_50_ value of 83.9 µM [54]. Five new antibacterial indole diketopiperazine alkaloids, 24,25-dihydroxyvariecolorin G (**133**), 25-hydroxyrubrumazine B (**134**), 22-chloro-25-hydroxyrubrumazine B (**135**), 25-hydroxyvariecolorin F (**136**), and 27-epi-aspechinulin D (**137**), were isolated from a fungal strain of deep-sea cold seep-derived *Aspergillus chevalieri* CS-122. Compound **134**, with hydroxyl groups at C-22 and C-23, exhibited broad-spectrum antibacterial activity against five tested bacterial strains (*V. harveyi*, *E. tarda*, *A. hydrophila*, *E. coli,* and *M. luteus*), with MIC values of 32, 16, 32, 16, and 32 µg/mL, respectively. Compound **137**, with hydroxyl groups at C-27 and C-28, exhibited broad-spectrum antibacterial activity against five tested bacterial strains (*V. harveyi*, *E. tarda*, *A. hydrophila*, *E. coli,* and *M. luteus*). Compound **136** showed moderate activity against the human pathogen *E. coli* and the aquatic bacterium *V. harveyi*, with the same MIC value of 32 µg/mL,. Compound **133** displayed significant inhibitory effects against *E. coli*, with an MIC value of 4 µg/mL, while compound **135** displayed noticeable inhibitory effects against *V. harveyi*, with an MIC value of 8 µg/mL [55]. Three new tripeptide derivatives, asterripeptides A–C (**138**–**140**), were isolated from mangrove *Kandelia candel*-derived fungus *Aspergillus terreus* LM.5.2. Compounds **138**–**140** contain a rare fungi cinnamic acid residue [56]. Two new compounds, 19*S*,20-epoxy-18-oxotryprostatin A (**141**) and 20-hydroxy-18-oxotryprostatin A (**142**), were isolated from the marine sediment-derived fungus *Aspergillus fumigatus* MF071. A genomic data analysis revealed the putative biosynthetic gene clusters ftm for fumitremorgins, pso for pseurotins, fga for fumigaclavines, and hel for helvolinic acid [57]. One new piperazinedione derivative, nigerpiperazine A (**143**), was isolated from the mangrove plant *Ceriops tagal* fungus *Aspergillus niger* JX-5. Compound **143** showed inhibitory activities against *Helicoverpa armigera* Hubner, with the IC_50_ value of 200 μg/mL [58]. Four new diketopiperazine-type alkaloids, oxepinamides H–K (**144**–**147**), were isolated from the culture broth extracts of the deep-sea-derived fungus *Aspergillus puniceus* SCSIO z021. Compounds **144**–**146** showed significant transcriptional activation on LXR*α,* with EC_50_ values of 15, 15, and 16 μM, respectively [59]. Two new diketopiperazine alkaloids, chevalinulins A (**148**) and B (**149**), containing an unprecedented spiro-[bicyclo [2.2.2]octane-diketopiperazine] skeleton, were isolated from the deep-sea cold-seep-derived fungus *Aspergillus chevalieri* CS-122. Their structures were determined by single-crystal X-ray diffraction, specific rotation (SR), and NMR calculations. The putative biosynthesis of **148** and **149** might start with the production of cyclo-*L*-Trp-*L*-Ala catalyzed by a nonribosomal peptide synthetase (NRPS) or by a cyclodipeptide synthase (CDPS), followed by monoprenylation catalyzed by a prenyltransferase to form preechuinulin. Compounds **148** and **149** both remarkably increased the number of intersegmental blood vessels (ISVs) in model zebrafish at the concentrations of 40 and 80 μg/mL, indicating that **148** and **149** exhibited significant proangiogenic activity. The above proangiogenic activities of **148** and **149** provide additional potential leads in the treatment of a series of diseases related to insufficient angiogenesis, such as ischemic heart disease, coronary artery disease, and stroke [60] (Figure 7).

### 2.3. Cyclopeptide Alkaloids

Cyclopeptides are macrocyclic compounds, the ring system of which consists of a hydroxystyrylamine moiety, an amino acid, and a *β*-hydroxy amino acid, and which is substituted with one or two additional units. A large number of natural cyclopeptides have been reported to be highly effective against different cancer cells, some of which are renowned for their clinical uses [61,62]. A total of 32 new cyclopeptide alkaloids were discovered from marine-derived *Aspergillus* species; among them, 12 compounds have novel skeleton structures (Figure 8, Figure 9 and Figure 10). Eleven of them showed cytotoxic, antibacterial activities, antifungal activities, anti-inflammatory, and pancreatic lipase-inhibitory activities.

A novel cyclic tripeptide, sclerotiotide M (**150**), was isolated from the culture of a marine-derived fungus, *Aspergillus ochraceopetaliformis* DSW-2 [63]. Four new peptides, JG002CPA (**151**), JG002CPB (**152**), FJ120DPA (**153**), and FJ120DPB (**154**), were isolated from the culture broths of the marine-derived fungi *Aspergillus allahabadii* JG002 and *A. ochraceopetaliformis* FJ120. Compound **152** exhibited the strongest inhibitory activity (IC_50_, 53.1 μM), better than the positive controls berberine chloride (IC_50_, 104.3 μM) and curcumin (IC_50_, 47.8 μM), and **152** also showed weak inhibition (IC_50_, 104.3 μM) of isocitrate lyase (ICL) derived from *C. albicans* [64]. Five new pentadepsipeptides, aspertides A–E (**155**–**159**), were isolated from the co-culture of mangrove *Sonneratia paracaseolaris-*derived fungus *Aspergillus tamarii* MA-21 and *Aspergillus insuetus* SD-512. Compounds **156**–**159** possessed uncommon amino acid residues, such as 3-hydroxyproline, 2,3-dihydroxyproline, or pipecolinic acid. Compounds **158** and **159** exhibited antibacterial activities against *E. tarda*, *V. alginolyticus*, *V. anguillarum*, *V. vulniffcus*, and *S. aureus*, with MIC values of 8–32 μg/mL, respectively. Compound **158** showed antibacterial activity against *E. tarda*, *V. alginolyticus*, *V. anguillarum*, and *V. vulniffcus,* with MIC values of 8, 16, 32, and 8 μg/mL, respectively. Compound **159** exhibited an inhibitory effect against *E. tarda* and *S. aureus,* with MIC values of 16 and 8 μg/mL, respectively [65]. Two new cyclohexadepsipeptides, japonamides A (**160**) and B (**161**), were isolated from the marine sponge-derived fungus *Aspergillus japonicus* MCCC 3A00261 based on molecular networking. Compounds **160** and **161** revealed synergistic antifungal activity against *Candida albicans*, with the same MIC value of 3.125 µM and FICI value of 0.03. The absence of toxicity to mammalian cells indicated the safety of the drug, which has important implications for the research and development of drugs. The above results suggest that the combination of **160** or **161** with antifungal drugs may be an effective anti-*C. albicans* regimen. Additionally, the results opened up a new method for cyclic peptides as synergistic antifungal active molecules in combination with fluconazole, ketoconazole, and rapamycin against resistant *C. albicans*. The underlying synergistic mechanism requires further exploration [66] (Figure 8).

Seven new cyclopentapeptides, pseudoviridinutans A–G (**162**–**168**), were isolated from the marine sediment-derived fungus *Aspergillus pseudoviridinutans* TW58-5 based on molecular networking. Compounds **162**–**168**, with a rare amino acid moiety, *O*,*β*-dimethyltyrosine, were observed for the first time from a marine-derived fungus. Compound **167**, displayed obvious inhibitory effects on NO production, stimulated by LPS at 20 μM, with no obvious cytotoxicity. Further, the dose study suggested that **167** dose-dependently inhibited NO production stimulated by LPS and compound **167** inhibited LPS-stimulated NO production in cultured murine macrophage RAW264.7 cells by reducing the expression of iNOS and NLRP3 [67]. Three new cyclohexapeptides, petrosamides A–C (**169**–**171**), were isolated from the marine sponge *Petrosia* sp.-derived fungus *Aspergillus* sp. 151304. Compounds **169**–**171** displayed significant and dose-dependent pancreatic lipase (PL)-inhibitory activities, with IC_50_ values of 7.6 ± 1.5, 1.8 ± 0.3, and 0.5 ± 0.1 μM, respectively. Further inhibition kinetics analyses showed that **171** inhibited PL in a noncompetitive manner, while molecular dynamics simulation revealed that it could bind to PL at the entrance of the catalytic pocket [68]. A new cyclic pentapeptide, cotteslosin C (**172**), was isolated from a co-culture of the sponge-associated fungus *Aspergillus versicolor* with *Bacillus subtilis* [69]. A new cyclic tetrapeptide, asperhiratide (**173**), was identified, obtained from the soft coral-derived fungus *Aspergillus hiratsukae* SCSIO 5Bn1003 [70]. One new cyclopeptide, versicolide A (**174**), was isolated from the seafloor-derived *Aspergillus versicolor* PS108-62 [71]. Three undescribed cyclic lipopeptides, maribasins C–E (**175**–**177**), were isolated from the marine gorgonian-associated fungus *Aspergillus* sp. SCSIO 41501. Compound **175** showed significant antifungal activity against five phytopathogenic fungal strains (*Fusarium oxysporum*, *Curvularia australiensis*, *Pyricularia oryzae*, *Alternaria solani,* and *Colletotrichum gloeosporioiles*), with MIC values of 25, 6.25, 50, 25, and 12.5 μg/disc, respectively. Compound **176** showed significant antifungal activity against *F. oxysporum*, *C. australiensis*, *P. oryzae*, *A. solani,* and *C. gloeosporioiles*, with MIC values of 12.5, 3.12, 12.5, 25, and 12.5 μg/disc, respectively. Compound **177** showed significant antifungal activity against *F. oxysporum*, *C. australiensis*, *P. oryzae*, *A. solani,* and *C. gloeosporioiles*, with MIC values of 50, 12.5, 50, 25, and 25 μg/disc, respectively. The structure–bioactivity relationship exhibited that the *β*-amino fatty acid chain could significantly affect the antifungal activity of this type of cyclic lipopeptides [21]. Three new aspochracin-type cyclic tripeptides, sclerotiotides M–O (**178**–**180**), were obtained from the fungus *Aspergillus insulicola* HDN151418, which was isolated from an unidentified Antarctica sponge. Compound **178** showed broad antimicrobial activity against a panel of pathogenic strains, including *B. cereus*, *Proteus species*, *Mycobacterium phlei*, *B. subtilis*, *V. parahemolyticus*, *E. tarda*, MRCNS, and MRSA, with MIC values of 3.13, 3.13, 3.13, 6.25, 3.13, 1.56, 12.5, and 25.0 µM, respectively. Compound **179** showed broad antimicrobial activity against *B. cereus*, *P. species*, *M. phlei*, *B. subtilis*, *V. parahemolyticus*, *E. tarda*, MRCNS, and MRSA, with MIC values of 6.25, 6.25, 12.5, 12.5, 6.25, 1.56, 25.0, and 25.0 µM, respectively [72]. A new *N*-methyl cyclic pentapeptide, caletasin (**181**), was isolated from the marine-facultative *Aspergillus* sp. MEXU 27854 from Caleta Bay, Mexico [73] (Figure 9).

### 2.4. Quinazoline Alkaloids

Quinazoline is a nitrogen-containing heterocyclic compound illustrated by a double-ring structure that contains a benzene ring system fused to pyrimidine at two adjacent carbon atoms, and has several pharmacological activities [74]. A total of 39 new quinoline alkaloids (**182**–**218**, including 2 pairs of racemates) were discovered from the marine-derived *Aspergillus* species. Among them, three compounds were found to have novel skeleton structures and fifteen compounds showed cytotoxic and antibacterial activities (Figure 10).

One new quinazoline alkaloid, chaetominine A (**182**), was isolated from the strain *Aspergillus fumigatus* MF029, which was obtained from a marine sponge, *Hymeniacidon perleve,* collected from the Bohai Sea, China [75]. Three new quinazoline-containing indole alkaloids, aspertoryadins H–J (**183**–**185**), were isolated from the marine-derived fungus *Aspergillus* sp. HNMF114 [76]. Two new quinazoline alkaloids, chaetominines A (**186**) and B (**187**), were isolated from marine sponge *Callyspongia* sp.-derived fungus *Aspergillus versicolour* SCSIO XWS04 F52. Compounds **186** and **187** showed cytotoxic activity against leukemia K562 and colon cancer cells SW1116, with IC_50_ ranging from 7.5 to 12.5 μM, and also exhibited significant protection against H1N1 virus-induced cytopathogenicity in MDCK cells, with IC_50_ values of 15.5 and 24.5 μM (oseltamivir as a positive control, IC_50_ 3.19 μM), respectively [77]. Three new 4-quinazolinone alkaloids, puniceloids E–G (**188**–**190**), were isolated from the culture of the marine-derived fungus *Aspergillus puniceus* FAHY0085. Compounds **189** and **190** showed the significant transcriptional activation of liver X receptor *α* with the same EC_50_ value of 1.7 μM, [36]. One new quinazolinone alkaloid, 2-(4-hydroxybenzyl)-4-(3-acetyl)quinazolin-one (**191**), was isolated from the seawater-derived fungus *Aspergillus sydowii* SW9. Compound **191** exhibited selective inhibitory activities against the human pathogenic bacteria *E. coli*, *S.aureus*, *S. epidermidis*, and *S. pneumoniae*, with MIC values of 16, 8.0, 4.0, and 16 µg/mL, respectively [78]. Seven new quinazoline-containing indole alkaloids, aspertoryadins A–G (**192**–**198**), were isolated from the *Sanguinolaria chinensis*-derived fungus *Aspergillus* sp. HNMF114. Compound **192** had a rarely aminosulfonyl group in its structure. Compounds **197** and **198** exhibited quorum-sensing-inhibitory activity against *Chromobacterium violaceum* CV026, with the same MIC values of 32 μg/well, respectively [79]. One new quinazoline alkaloid, 2-*epi*-tryptoquivaline F (**199**), was isolated from the marine-derived fungus *Aspergillus fumigatus* H22 [17]. A new quinazoline alkaloid, protuboxepin K (**200**), was isolated from the culture broth of the marine-derived fungal strain *Aspergillus* sp. BFM-0085, collected from a sediment sample of Tokyo Bay. Compound **200** inhibited bone morphogenetic protein (BMP)-induced alkaline phosphatase activity with an IC_50_ value of 4.7 μM in mutant BMP receptor-carrying C2C12 (R206H) cells [80]. Five new quinazolinone alkaloids, felicarnezolines A–E (**201**–**205**), were isolated from the co-culture of marine-derived fungi *Amphichorda* sp. KMM 4639 and *Aspergillus carneus* KMM 4638. Compounds **203**–**205** showed cytotoxic activity against human breast cancer MCF-7 cells with IC_50_ values of 92.5 ± 3.1, 68.7 ± 1.6, and 86.3 ± 2.3 μM, respectively. Compound **204** protected rat cardiomyocytes H9c2 and human neuroblastoma SH-SY5Y cells against CoCl_2_-induced damage, with the IC_50_ value of 72.9 ± 2.8 μM, showing a good protective effect in hypoxiamimic conditions via antioxidant pathways. Compound **205** showed cytotoxic effects against human prostate cancer PC-3 cells, with an IC_50_ value of 83.8 ± 5.5 μM [81]. One new quinazoline, (−)-isoversicomide A (**206**), was isolated from the fungus *Aspergillus versicolor* PS108-62 [71]. One new quinazoline alkaloid, 29-hydroxyfumiquinazoline C (**207**), was isolated and identified from the deep-sea sediment-derived fungus *Aspergillus fumigatus* SD-406 [20]. Two new alkaloid racemates, (±)-17-hydroxybrevianamide N (**208**) and (±)-N1-methyl-17-hydroxybrevianamide N (**209**), featuring a rare *O*-hydroxyphenylalanine residue and an imide subunit, were isolated from a soft-coral *Sinularia* sp.-derived fungus *Aspergillus* sp. CHNSCLM-0151. Simultaneously, their structures, including their absolute configurations, were elucidated on the basis of comprehensive spectroscopic analysis, ECD calculations, and X-ray diffraction data. Interestingly, the basic solution promotes the racemization of (+)-**208** and (−)**-208**, whereas acidic solution suppresses the transformation [82]. Four new 4-quinazolinone alkaloids, puniceloids A–D (**210**–**213**), were isolated from the culture broth extracts of the deep-sea sediment-derived fungus *Aspergillus puniceus* SCSIO z021. Compounds **210**–**213** showed significant transcriptional activation on LXRα, with EC_50_ values of 5.1, 5.3, 1.7, and 1.7 μM, respectively [59]. One new quinazolinone alkaloid, novobenzomalvin D (**214**), was isolated from *Aspergillus terreus* SCAU011, a fungus from the rhizosphere sediment of a mangrove plant *Rhizophora stylosa*. Compound **214** exhibited better COX-2-inhibitory activity (inhibition rate of 91.1%) than the positive control celecoxib (56.7%) at 20 nM [83]. A new alkaloid, tryptoquivaline Y (**215**), was purified from a Hawaiian beach soil-derived fungus *Aspergillus felis* FM324 [84]. A new fumiquinazoline-type alkaloid, 2-methyl-versiquinazoline C (**216**), was isolated from *Aspergillus flavipes* PJ03-11 [85]. Two new glucosidated indole-containing quinazoline alkaloids, fumigatosides G (**217**) and H (**218**), were isolated from mangrove-derived fungus *Aspergillus fumigatus* SAl12 [86] (Figure 10).

### 2.5. Isoquinoline Alkaloids

Isoquinoline alkaloids, a large class of natural products, are mostly found in plants and also found in the extracts of bacterial and fungal cultures. They possess a broad range of biological activities, including antimicrobial, antitumor, antileukemic, and anti-inflammatory properties [87]. A total of 17 new isoquinoline alkaloids were discovered from marine-derived *Aspergillus* species (Figure 11), and 5 of them showed cytotoxic activities, antibacterial inhibitory activity, and inhibitory activity against protein tyrosine phosphatase CD45.

Sixteen new isoquinoline alkaloids, puniceusines A–N (**221**–**234**), puniceusine O (**219**), and (±)-puniceusine P (**220**), were isolated from the extracts of a deep-sea sediment-derived fungus, *Aspergillus puniceus* SCSIO z021. Compounds **223** and **224** showed selective inhibitory activity against protein tyrosine phosphatase CD45 with IC_50_ values of 8.4 and 5.6 µM, respectively, and **224** also showed moderate cytotoxicity towards human lung adenocarcinoma cell line H1975 with an IC_50_ value of 11.0 µM. Compound **234** showed medium antibacterial activity towards *S. aureus*, methicillin-resistant *S. aureus* (MRSA), and *E. coli*, with MIC values of 100 µg/mL, and compound **224** could inhibit the growth of *E. coli* with an MIC value of 100 µg/mL. The structure–activity relationship of **221**–**234** revealed that the substituents at C-7 of the isoquinoline nucleus could greatly affect their bioactivity [88,89]. One new alkaloid, 2-(quinoline-8-carboxamido)benzoic acid (**235**), was isolated from the deep-sea sediment-derived fungus *Aspergillus* sp. SCSIO06786 [90] (Figure 11).

### 2.6. Pyrrolidine Alkaloids

Pyrrolidine alkaloids have been shown to possess several important biological activities, including antioxidant, anti-inflammatory, antibacterial, antifungal, antiparasitic and anthelmintic, anticancer, anti-hyperglycemic, organ-protective, and neuropharmacological activities [91]. A total of 22 new pyrrolidine alkaloids (**236**–**256**, including 1 pair of racemates) were discovered from marine-derived *Aspergillus* species. Three compounds have novel skeleton structures (Figure 12) and nine of them showed cytotoxic activities, antibacterial activities, antifungal activities, and anti-inflammatory activities.

Two rare tetracyclic skeleton alkaloids, perinadines B and C (**236** and **237**), the first reported derivatives of a rare type of tetracyclic alkaloid, perinadine A, were isolated as mixtures of epimers from the marine sponge *Haliclona* sp.-derived fungus *Aspergillus* sp. LS116, driven by molecular networking. Compounds **236** and **237** showed moderate antibacterial activity against *B. subtilis* with MIC values of 32 and 64 μg/mL, respectively [92]. One new pyrrolidine alkaloid, ochraceopetalin (**238**), a mixed-biogenetic salt compound, with a sulfonated diphenylether–aminol–amino acid ester guanidinium salt of an unprecedented structural class, was isolated from a marine sediment-derived fungus *Aspergillus ochraceopetaliformis* FJ120. Compound **238** exhibited significant cytotoxicity against K562 and A549 cells, with IC_50_ values of 9.5 and 6.8 µM, respectively [93]. Three new tricyclic cyclopiazonic acid-related alkaloids, asperorydines N–P (**239**–**241**), were isolated and characterized from the fungus *Aspergillus flavus* SCSIO F025, derived from the deep-sea sediments of South China Sea [94]. One new pyrrolidine alkaloid, variotin B (**242**), was isolated from the deep-sea shrimp fungus *Aspergillus unguis* IV17-109. Compound **242** showed moderate anti-inflammatory activity, with an IC_50_ value of 20.0 µM [95]. One new pyrrolidine alkaloid, (*E*)-6-hydroxy-5-(1-propenyl)-1,2-dihydropyrano [3,2-b]pyrrole-3,7-dione (**243**), was obtained from the rhizosphere soil of mangrove *Bruguiera gymnorrhiza* (L.)-derived fungus *Aspergillus* sp. DM94 [96]. Two new spiro-heterocyclic *γ*-lactam derivatives, cephalimysins M (**244**) and N (**245**), were isolated from the fermentation cultures of the marine sediment-derived fungus *Aspergillus fumigatus* CUGBMF170049 [97]. Five new azaspirene derivatives, azaspirenes A–E **(246**–**250**), were isolated from the marine mud-derived endophytic fungus *Aspergillus micronesiensis* NF666, and a plausible biosynthetic pathway of azaspirenes was proposed [98]. One new pyrrolidine alkaloid, 10*R*-15-methylpseurotin A (**251**), was isolated from the deep-sea sediment-derived fungus *Aspergillus fumigatus* SD-406. Compound **251** exhibited moderate activity against the plant-pathogenic fungi *F. graminearum* Schw., with an MIC value of 16 µg/mL [20]. A new alkaloid, pseurotin I (**252**), was purified from a fungal strain *Aspergillus felis* FM324, which was isolated from a Hawaiian beach soil sample. Compound **252** inhibited NF-κB with an IC_50_ value of 30.9 μM, respectively [84]. Two pyrrolinone-fused benzoazepine alkaloids, (±)-asperazepanones A (**253**) and B (**254**), were isolated from the coral-derived *Aspergillus candidus* fungus. Compound **254** showed obviously inhibitory activity against nitric oxide (NO) production, with an inhibition rate of 43 ± 4% at the concentration of 1 μM. The levels of TNF-*α* (*p* < 0.0001) and IL-6 (*p* < 0.01) significantly decreased, with 40 ± 2% and 77 ± 7% inhibition rates, respectively, at the concentration of 0.1 μM [99]. A new oxygenated tricyclic cyclopiazonic acid alkaloid, asperorydine Q (**255**), was isolated from the coral-associated *Aspergillus flavus* GXIMD 02503. Compound **255** exhibited moderate inhibitory activity against NF-κB activation, with an IC_50_ value of 14.1 ± 1.5 μM [100]. A new alkaloid, pyranonigrin L (**256**), was isolated from mangrove endophytic fungus *Aspergillus fumigatus* SAS10 [101] (Figure 12).

### 2.7. Other Heterocyclic Alkaloids

A total of 37 new other heterocyclic alkaloids were discovered from the marine-derived *Aspergillus* species (Figure 13, Figure 14 and Figure 15); 11 compounds have novel skeleton structures and 12 of them showed cytotoxic activities, antibacterial activities, antifungal activities, *α-*glucosidase-inhibitory activities, PTP1B inhibitory activities, and so on.

Six new benzoic acid-containing alkaloids, asperalin A–F (**257**–**262**), were isolated and identified from a seagrass *Enhalus acoroides*-derived *Aspergillus alabamensis* SYSU-6778. Compounds **259** and **260** showed strong activity against *S. aureus*, *S. iniae*, and *S. parauberis*, with MIC values of 10.1, 5.0, and 10.1 μM, respectively. Compound **261**, an *N*-alkylated product of **260**, exhibited the strongest inhibitory effects against *S. iniae*, with an MIC value of 2.2 μM. Compound **262**, as a new natural bactericide, showed moderate to potent inhibitory activity against Gram-negative bacterium *E. ictalurid* and four Gram-positive bacteria (*S. Iniae, S. aureus, S. parauberis* and *Bacillus subtilis*), with MIC values of 10.9, 43.6, 21.8, 87.3, and 21.8 μM, respectively [102]. A new aflaquinolone, 22-epi-aflaquinolone B (**263**), was isolated from a co-culture of the sponge-associated fungus *Aspergillus versicolor* with *B. subtilis* [69]. Three new quinolone alkaloids, aspergillspins C–E (**264**–**266**), were isolated from the marine gorgonian *Melitodes squamata*-derived fungus *Aspergillus* sp. SCSIO 41501 [23]. Two new alkaloids, citriquinolinones A (**267**) and B (**268**), were isolated from the deep-sea whale *Mesoplodon densirostris*-derived fungus *Aspergillus versicolor* 170217. Compounds **267** and **268,** featuring a unique isoquinolinone-embedded citrinin scaffold, represented the first examples of citrinin-isoquinolinone hybrids [103] (Figure 13).

A new benzodiazepine alkaloid, circumdatin M (**269**), with a rare pyrimidone-4-pyrone moiety, was isolated from a Hawaiian marine fungus *Aspergillus* sp. FM242. The structure and absolute configuration of **269** was determined through an analysis of HRMS and NMR spectroscopic data, DP4+ NMR calculations, and CD calculations [104]. Two new nucleoside derivatives, kipukasins M (**270**) and N (**271**), were obtained from the sea mud-derived fungus *Aspergillus versicolor* TJ-LHQ-AV507. Interestingly, intramolecular transesterification occurred in **270** and **271**, which existed as a pair of inseparable regioisomers [105]. One new pteridine alkaloid, asperpteridinate A (**272**), was isolated from the seawater sample-derived fungus *Aspergillus austroafricanus* Y32-2 [13]. A new alkaloid, pyripyropene U (**273**), was obtained from the marine sponge-derived fungus *Aspergillus* sp. SCSIO41420 [106]. A new alkaloid, aspernigrin E (**274**), was isolated from mangrove endophytic fungus *Aspergillus fumigatus* SAS10 [101]. One new azaphthalide derivative, (*S*)-3-hydroxy-2,7-dimethylfuro [3,4-b]pyridin-5(7*H*)-one (**275**), was isolated from the coral-derived fungus *Aspergillus* sp. SCSIO41405 [107]. A new alkaloid, asperalumazine A (**276**), was isolated and identified from a seagrass-derived *Aspergillus alabamensis* SYSU-6778. Compound **276** represented the first example of a lumazine derivative directly coupled to a benzoic acid moiety via a hydroxymethyl group [102]. Three new polypropionate derivatives, fiscpropionates D–E (**277**–**278**), representing the first examples of polypropionate derivatives containing a 3-hydroxypiperidin-2-one as part of an imide linkage, were obtained from the deep-sea-derived fungus *Aspergillus fischeri* FS452. Compound **277** exhibited significant inhibitory activities against *Mycobacterium tuberculosis* protein tyrosine phosphatase B (*M*ptpB), with an IC_50_ value of 11 μM [108] (Figure 14).

One new compound, rhizoaspergillin A (**279**), was isolated from the mangrove *Rhizophora mucronata* endophytic fungus *Aspergillus* sp. A1E3. From the perspective of biosynthesis, **279** could originate from the combined assembly of three building blocks, viz., orsellinic acid, *β*-*D*-ribofuranose, and *L*-glutamine. It is an unprecedented alkaloid-N-oxide involving the biosynthetic pathways of polyketides, pentose, and amino acids [109]. One new acremolin-type alkaloid, acremolin D (**280**), was isolated from the deep-sea-derived fungus *Aspergillus sydowii* MCCC 3A00324. Compound **280** showed inhibitory effects against Hela-S3 and K562 cell lines, with inhibition rates of 30.6% and 25.1%, respectively, at the concentration of 20 μM [110]. A new oxidized phomaligol derivative, phomaligol H (**281**), was isolated from the culture of the fungus *Aspergillus flavus* BB1; the fungus was isolated from the marine shellfish *Meretrix meretrix*, collected on Hailing Island. Compound **281** demonstrated cytotoxic activity against the A549 cell line, with an IC_50_ value of 65.53 μM [111]. Four undescribed pyrazinopyrimidine-type alkaloids, including three natural products, pyrasplorines A–C (**282**–**284**), and an artifact deg-pyrasplorine B (**285**), as well as a biogenetically related versicoloid A (**286**), were discovered from the extract of a mangrove *Thespesia populnea*-derived fungus *Apergillus verisicolor* HDN11-84. Compound **282** had a unique spiral-type skeleton (composed of a cyclopentenone ring with a pyrazino [1,2-a] pyrimidine core), which is unprecedented in pyrazinopyrimidine-type alkaloids [112]. A new compound, penilumamide K (**287**), was obtained from the deep-sea-derived fungus *Aspergillus* sp. SCSIO 41029. Compound **287** represented the first lumazine peptide reported from deep-sea-derived fungus. Compound **287** had significant potency against *α*-glucosidase, with an IC_50_ value of 18.61 μΜ [113]. One new compound (6-benzyl-1-isopentyl-4-oxo-1,4-dihydropyridin-3-yl)-carboxamide (**288**) was obtained from a mangrove *Bruguiera gymnorrhiza* (L.)-derived fungus strain of *Aspergillus* sp. DM94 [96]. Three unusual chlorinated metabolites, flavipesides A–C (**289**–**291**), were isolated from the marine sponge-derived fungus *Aspergillus flavipes* 164013. Their structures were determined by spectroscopic data analysis, and absolute configurations were assigned through single-crystal X-ray diffraction with ECD spectral analysis. Compounds **289**–**291** represented a new structural family of PKS-NRPS hybrid metabolites with an uncommon assembly of structural scaffolds and functional groups. Each of them was composed of a chlorinated, methylated, and hydroxylated xanthone and an aminoethyl-modified pyrazol, as well as a methylated dipeptide. Compound **289**–**291** exhibited potent inhibitory activity against PL, with IC_50_ values of 0.23 ± 0.03, 0.07 ± 0.01, and 0.14 ± 0.02 μM, respectively. They were 6–21 times more potent than the positive control kaempferol with an IC_50_ value of 1.50 ± 0.21 μM. A preliminary structure–activity relationship analysis revealed that the inhibitory activity potency of **289**–**291** was influenced by the variations in chlorination positions on the xanthone substructure: 2-chloridated > 4-chloridated > 2,4-dichloridated. These isolates were discovered to be a new family of PL inhibitors with therapeutic potential to prevent hyperlipidemia and obesity [114]. Two new nucleoside derivatives, kipukasins K (**292**) and L (**293**), were obtained from the gorgonian *Dichotella gemmacea*-derived fungus *Aspergillus versicolor* XS-20090066-based molecular networking; the fungus was induced by chemical epigenetic manipulation with a combination of 100 µM SAHA and 100 µM 5-Aza. Chemical epigenetic manipulation should be a feasible and effective strategy to trigger the production of bioactive secondary metabolites from marine-derived fungi [115] (Figure 15).

## 3. Conclusions

**Figure 16:**
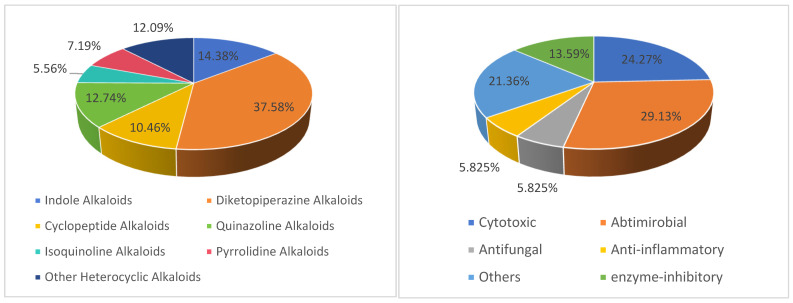
Structural diversity (**left**) and bioactivities (**right**) of new nitrogen heterocycles isolated from *Aspergillu*s pp. that were discovered from January 2019 to January 2024.

In this review, the sources, structural diversity, and biological activity of secondary metabolites from marine-derived *Aspergillus* species are summarized, covering the period between January 2019 and January 2024. A total of 306 new nitrogen heterocycles were obtained from the genus *Aspergillus*. Remarkably, among them, 52 compounds had novel skeleton structures. One hundred and three compounds, along with their biological activities, producing strains, and habitats, are summarized in Table 1. The structure type and the bioactivity distribution of the new nitrogen heterocycles isolated from *Aspergillu*s pp. are also shown in Figure 16.

The chemical structures of the 306 new secondary metabolites from Aspergillus species can mainly be classified into six types, including 44 indole alkaloids (including 1 pair of racemates), 115 diketopiperazine alkaloids (including 9 pairs of racemates), 32 cyclopeptide alkaloids, 39 quinazoline alkaloids (including 2 pairs of racemates), 17 isoquinoline alkaloids, 22 pyrrolidine alkaloids (including 1 pair of racemates), and 37 other heterocyclic alkaloids. However, among these 306 new compounds, diketopiperazine alkaloids accounted for 37.58%, while indole alkaloids, cyclopeptide alkaloids, quinazoline alkaloids, isoquinoline alkaloids, pyrrolidine alkaloids, and other heterocyclic alkaloids accounted for 14.38%, 10.46%, 12.74%, and 5.56%, 7.19%, and 12.09%, respectively (Figure 16). Due to the high salt levels of the marine environment, some nitrogen-containing secondary metabolites from marine-derived *Aspergillus* species contain halogen atoms, such as compounds **33**, **37** and **259**–**262**.

Moreover, it is worth noting that nearly 33.66% (103 compounds) showed broad-spectrum biological activities, including antimicrobial activities (30 compounds), cytotoxic activities (25 compounds), enzyme-inhibitory activities (14 compounds), antifungal activities (6 compounds), anti-inflammatory activities (6 compounds), and other activities (22 compounds). Notably, cytotoxic (24.27%), antimicrobial (29.13%), and other activities (21.36%) represent the top three bioactivities (Figure 16). It is important to highlight that 21 compounds exhibit potent activities. For example a, new indole alkaloid, sclerotiamide C (**13**), showed significant inhibitory activity against HeLa, A549, HepG2, and SMMC7721 cell lines, with IC_50_ values of 1.7 ± 0.1, 1.6 ± 0.1, 1.8 ± 0.1, and 1.5 ± 0.1 μM, respectively, and **13** could induce apoptosis in HeLa cells by arresting the cell cycle, activating ROS production, and regulating apoptosis-related proteins in the MAPK signaling pathway, suggesting that **13** is a potential lead for further development as an anti-cervical tumor agent. A new diketopiperazine alkaloid, waikikiamide A (**97**), showed antiproliferative activity against cell lines (HT1080, PC3, Jurkat, A2780S) with IC_50_ values of 0.519, 1.855, 0.62, and 0.78 μM, respectively. New peptides, JG002CPB (**152**), exhibited the strongest inhibitory activity (IC_50_ = 53.1 μM), better than that of the positive controls berberine chloride (IC_50_ = 104.3 μM). One new quinazolinone alkaloid, novobenzomalvin D (**214**), exhibited better COX-2-inhibitory activity (an inhibition rate of 91.1%) than the positive control celecoxib (56.7%) at 20 nM.

**Figure 17:**
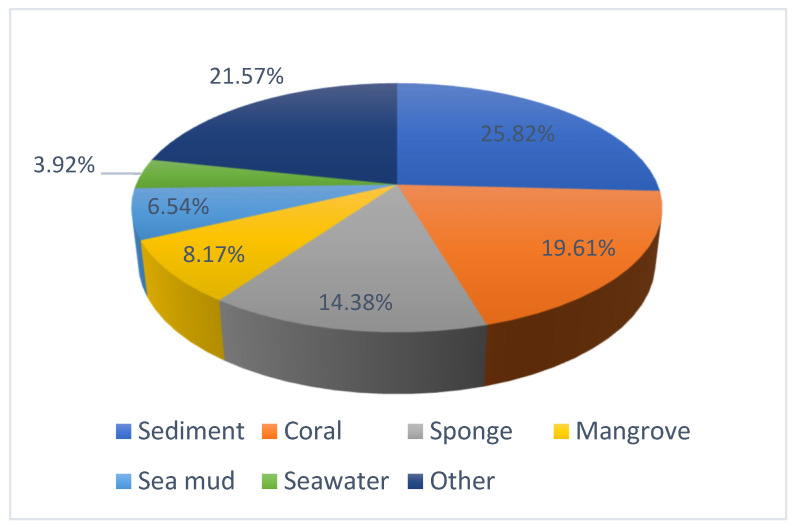
The proportion of *Aspergillus* species from different marine sources.

Additionally, the samples were obtained from various environments: 25.82% from sediment, 19.61% from coral, 14.38% from sponge, 8.17% from mangrove, 6.54% from sea mud, 3.92% from seawater, and 21.57% from shellfish, seagrass, and other marine resources. Most significantly, 25.82% originated from marine sediment samples (Figure 17).

In summary, *Aspergillus* species have been proven to be an important source of novel structures and diverse secondary metabolites with a broad range of biological activities, revealing their great untapped potential in medicinal and agrochemical applications. However, for most of the bioactive secondary metabolites, the lack of deep pharmacological mechanisms limited their applications. We should focus on the pharmacological mechanisms, pharmacokinetics, medicinal chemistry, biosynthesis, etc., to promote the development of innovative drugs in future studies.

## Figures and Tables

**Table 1:** The producing strains, habitats, Genbank accession number, and bioactivities of secondary metabolites from marine-derived *Aspergillus* species.

Compounds	Producing Strains	Habitats	Genbank Accession Number	Bioactivities	Refs.
Aculeaquamide A (**1**)	*A. aculeatinus* WHF0198	Deep-sea sediment, South China Sea	–	IC_50_ (cytotoxicity) 3.3 μM	[10]
Asterriquinone F (**2**)	*A. terreus* LM.1.5	Leaves of an unidentified mangrove tree, Vietnam, South China Sea	MN788658.1	–	[11]
Asperdiazapinone G (**3**)	*Aspergillus* sp. WHUF03110	Mangrove soil, Yalong Bay, at Sanya, Hainan, China	MZ661122	–	[12]
Di-6-hydroxydeoxybrevianamide E (**4**)	*A. austroafricanus* Y32-2	Seawater, Indian Ocean	MK267449	–	[13]
Dinotoamide J (**5**)	*A. austroafricanus* Y32-2	Seawater, Indian Ocean	MK267449	Pro-angiogenic activity	[13]
				at concentration of 70 µg/mL	
Penerpene O (**6**)	*Aspergillus* sp. ZF-104	Marine soft coral, Haikou Bay, Hainan province, China	OM320573	IC_50_ (PTP1B-inhibitory activities) 17.7 ± 0.7 μM	[14]
Penerpenes Q-R (**7**–**8**)	*Aspergillus* sp. ZF-104	Marine soft coral, Haikou Bay, Hainan province, China	OM320573	–	[14]
Penerpenes T (**9**)	*Aspergillus* sp. ZF-104	Marine soft coral, Haikou Bay, Hainan province, China	OM320573	–	[14]
Penerpene U (**10**)	*Aspergillus* sp. ZF-104	Marine soft coral, Haikou Bay, Hainan province, China	OM320573	IC_50_ (PTP1B-inhibitory activities)	[14]
				28.1 ± 2.2 μM	
Penerpene V (**11**)	*Aspergillus* sp. ZF-104	Marine soft coral, Haikou Bay, Hainan province, China	OM320573	–	[14]
*O*-*β*-*D*-glucopyranosyl ester (**12**)	*A. fumigatus* M580	Sea cucumber, Co To-Thanh Lan island, Vietnam	MW015802	–	[15]
Sclerotiamide C (**13**)	*A. sclerotiorum* LZDX-33-4	Marine gorgonian, South China Sea	OK012383.1	IC_50_ (cytotoxicity) 1.5–1.8 μM	[16]
Sclerotiamides D-E (**14**–**15**)	*A. sclerotiorum* LZDX-33-4	Marine gorgonian, South China Sea	OK012383.1	–	[16]
Sclerotiamide F (**16**)	*A. sclerotiorum* LZDX-33-4	Marine gorgonian, South China Sea	OK012383.1	IC_50_ (cytotoxicity) 0.05–0.07 μM	[16]
Sclerotiamides G-H (**17**–**18**)	*A. sclerotiorum* LZDX-33-4	Marine gorgonian, South China Sea	OK012383.1	–	[16]
Fumindoline A–C (**19**–**21**)	*A. fumigatus* H22	Seawater, Western Pacific	NRRL 163 s	–	[17]
Aspercarbolines A–B (**22**–**23**)	*Aspergillus* sp. XBB-4	Inner tissue of geoduck *Panopea abbreviate*, South China Sea	MK863524	–	[18]
Aspercarboline C (**24**)	*Aspergillus.* sp. XBB-4	Inner tissue of geoduck *Panopea abbreviate*, South China Sea	MK863524	IC_50_ (cytotoxicity) 16.29–50.85 μM	[18]
Flavonoid A (**25**)	*A. flavipes* DS720	Deep seawater, Mariana Trench	ON340751	Inhibition rates of (cytotoxicity) (90.83 ± 3.31)%–(99.49 ± 0.50)%, at the concentration of 20 µM	[19]
Flavonoid B (**26**)	*A. flavipes* DS720	Deep seawater, the Mariana Trench	ON340751	–	[19]
Secofumitremorgins A and B (**27a** and **27b**)	*A. fumigatus* SD-406	Deep-sea sediment, the East China Sea	MT635279	MIC (antimicrobial) 4–64 µg/mL	[20]
Aspergillipeptides H–I (**28**–**29**)	*Aspergillus* sp. SCSIO 41501	Marine gorgonian *Melitodes squamata* Nutting, the South China Sea, Sanya, Hainan	JN851015	–	[21]
Ascandinines A–B (**30**–**31**)	*A. candidus* HDN15-152	Sponge, Pulitzer bay, Antarctica	MH430037	–	[22]
Ascandinine C (**32**)	*A. candidus* HDN15-152	Sponge, Pulitzer bay, Antarctica	MH430037	IC_50_ (anti-influenza virus) 26 μM	[22]
Ascandinine D (**33**)	*A. candidus* HDN15-152	Sponge, Pulitzer bay, Antarctica	MH430037	IC_50_ (cytotoxicity) 7.8 μM	[22]
Aspergillspins A-B (**34**–**35**)	*Aspergillus* sp. SCSIO 41501	Marine gorgonian *Melitodes squamata* Nutting, the South China Sea, Sanya, Hainan	JN851015	–	[23]
Asterriquinone I (**36**)	*Aspergillus* sp. SCSIO 41018	Sponge, Xuwen, Guangdong Province	MH109740.1	IC_50_ (cytotoxicity)	[24]
				17.9 ± 0.62–29.2 ± 0.32 μM	
Asterriquinone J (**37**)	*Aspergillus* sp. SCSIO 41018	Sponge, Xuwen, Guangdong Province	MH109740.1	IC_50_ (cytotoxicity)	[24]
				8.5 ± 0.17–18.7 ± 0.45 μM	
Asterriquinone K (**38**)	*Aspergillus* sp. SCSIO 41018	Sponge, Xuwen, Guangdong Province	MH109740.1	IC_50_ (cytotoxicity)	[24]
				13.0 ± 0.36–26.2 ± 0.13 μM	
Asterriquinols G–I (**39**–**41**)	*Aspergillus* sp. SCSIO 41018	Sponge, Xuwen, Guangdong Province	MH109740.1	–	[24]
Aspergillamides C-D (**42**–**43**)	*A. terreus* SCSIO 41008	Marine sponge *Callyspongia* sp., Xuwen County, Guangdong Province, China	MF536093	–	[25]
(±)-7,8-epoxy-brevianamide Q ((±)-**44**)	*A.* versicolor MF180151	Marine sediment, Bohai Sea, China.	MK680178	–	[28]
(±)-8-hydroxy-brevianamide R ((±)-**45**)	*A.* versicolor MF180151	Marine sediment, Bohai Sea, China.	MK680178	–	[28]
(±)-8-epihydroxy-brevianamide R ((±)-**46**)	*A.* versicolor MF180151	Marine sediment, Bohai Sea, China.	MK680178	–	[28]
(-)-5-isopentenyl-cryptoechinuline D (**47a**)	*A. ruber* TX-M4-1	Marine moss, Weizhou Island	OL989330	IC_50_ (inhibits TrxR activity)	[29]
				6.2 μM	
(+)-5-isopentenyl-cryptoechinuline D (**47b**)	*A. ruber* TX-M4-1	Marine moss, Weizhou Island	OL989330	–	[29]
(±)-dibrevianamide Q1 ((±)-**48**)	*Aspergillus* sp. ZA-01	Marine sediment, Bohai Sea	–	IC_50_ (anti-H1N1 virus activity, (+)-**44**) 12.6 μM; MIC (anti-bacterium) 10.2 μg/mL	[30]
(±)-dibrevianamide Q2 ((±)-**49**)	*Aspergillus* sp. ZA-01	Marine sediment, Bohai Sea	–	IC_50_ (anti-H1N1 virus activity, (−)-**45**) 19.5 μM	[30]
Versicolamide C (**50**)	*Aspergillus* sp. SCSIO 41036	Soft coral, Beihai, Guangxi, China	OM441922	–	[31]
Emestrins L-M (**51**–**52**)	*A. terreus* RA2905	Sea hare *Aplysia pulmonica*, Weizhou, South China Sea	MK611650	–	[32]
(±)-brevianamide Z ((±)-**53**)	*A. versicolor* HBU-7	Sea mud, Bohai, China	KY814754	–	[33]
(±)-brevianamide Z1 ((±)-**54**)	*A. versicolor* HBU-7	Sea mud, Bohai, China	KY814754	–	[33]
Pyranamides A-D (**55**–**58**)	*A. versicolor* SCSIO 41016	Marine sponge Callyspongia sp., Xuwen County, Guangdong Province, China	MH244341	–	[34]
Secopyranamide C (**59**)	*A. versicolor* SCSIO 41016	Marine sponge Callyspongia sp., Xuwen County, Guangdong Province, China	MH244341	–	[34]
Protuboxepins F-J (**60**–**64**)	*A. versicolor* SCSIO 41016	Marine sponge Callyspongia sp., Xuwen County, Guangdong Province, China	MH244341	–	[34]
Sclerotioloid A (**65**)	*A. sclerotiorum* ST0501	Sponge, Guangdong, China	MT534582	–	[35]
Sclerotioloid C (**66**)	*A. sclerotiorum* ST0501	Sponge, Guangdong, China	MT534582	–	[35]
Oxepinamide L (**67**)	*A. puniceus* FAHY0085	Marine coral, South China Sea	OQ825098	–	[36]
Asperindopiperazines A–C (**68**–**70**)	*Aspergillus* sp. SY2601	Mariana-Trench sediments	OR646740	–	[37]
12*β*,13*β*-hydroxy-asperfumigatin (**71**)	*A. fumigatus* H22	Seawater, Western Pacific	NRRL 163 s	–	[17]
(+)- and (-)-brevianamide X ((±)-**72**)	*A. versicolor* OUCMDZ-2738	*Enteromorpha prolifera*, Shilaoren beach, Qingdao, China	MH150818	–	[38]
Asperflocin (**73**)	*A. versicolor* 16F-11	Sponge, Yongxing Island, South China Sea, China	KM605199	IC_50_ (cytotoxicity) 10.29 ± 2.37 μM	[39]
Aspechinulins A-B (**74**–**75**)	*Aspergillus* sp. FS445	Deep-Sea sediment, Indian Ocean	MW386823	–	[40]
Aspechinulin C (**76**)	*Aspergillus* sp. FS445	Deep-Sea sediment, Indian Ocean	MW386823	IC_50_ (inhibition against NO production) 23.7 μM	[40]
Aspechinulin D (**77**)	*Aspergillus* sp. FS445	Deep-Sea sediment, Indian Ocean	MW386823	–	[40]
5-prenylcryptoechinulin A (**78**)	*A. chevalieri* MCCC M23426	Deep-Sea	NR_135340	Inhibition rate (antibacterial) of over 90% at the concentration of 250 µM	[41]
9-*epi*-didehydroechinulin (**79**)	*A. chevalieri* MCCC M23426	Deep-Sea	NR_135340	–	[41]
Asperdione A (**80**)	*Aspergillus* sp. XBB-4	Inner tissue of geoduck *Panopea abbreviate*, South China Sea	MK863524	IC_50_ (cytotoxicity) 10.72–22.00 μM	[18]
(+) and (-)19-epi-sclerotiamide	*A. versicolor* CGF 9-1-2	Soft coral, South China Sea	MG827180.1	–	[42]
(**81** and **82**)					
Didethio-11*α*-methylthioemestrin (**83**)	*A. nidulans* SD-531	Deep-Sea cold-seep sediment, South China Sea	MN901610.1	–	[43]
7′-*epi*-didethio-11α-methylthioemestrin (**84**)	*A. nidulans* SD-531	Deep-Sea cold-seep sediment, South China Sea	MN901610.1	IC_50_ (antimicrobial) 0.5–16.0 μM	[43]
2′′-desmethyl-MPC1001F (**85**)	*A. nidulans* SD-531	Deep-Sea cold-seep sediment, South China Sea	MN901610.1	IC_50_ (cytotoxicity) 8.0 μM	[43]
Asperthrins E–F (**86**–**87**)	*Aspergillus* sp. YJ191021	Soil, Zhoushan, Zhejiang, China	–	–	[44]
Asperthrin A (**88**)	*Aspergillus* sp. YJ191021	Soil, Zhoushan, Zhejiang, China	–	MIC (antimicrobial) 8–25 µg/mL; IC_50_ (anti-inflammatory) 1.46 ± 0.21 µM	[44]
Asperthrins B–D (**89**–**91**)	*Aspergillus* sp. YJ191021	Soil, Zhoushan, Zhejiang, China	–	–	[44]
Stereoisomers (**92**–**93**)	*Aspergillus* sp. Z3	Marine isopod *Ligia exotica*, Dinghai, Zhoushan, Zhejiang, China	–	–	[45]
3-hydroxyprotuboxepin K (**94**)	*A. creber* EN-602	Red algal *Rhodomela* *confervoides*, Qingdao, China	MW186501	IC_50_ (enzyme-inhibitory activity) 22.4 μM	[46]
3,15-dehydroprotuboxepin K (**95**)	*A. creber* EN-602	Red algal *Rhodomela* *confervoides*, Qingdao, China	MW186501	MIC (antimicrobial) 8–64 µM	[46]
Versiamide A (**96**)	*A. creber* EN-602	Red algal *Rhodomela* *confervoides*, Qingdao, China	MW186501	MIC (antimicrobial) 16–64 µM;	[46]
Waikikiamide A (**97**)	*Aspergillus* sp. FM242	soil, Waikiki beach of Oahu, Honolulu, Hawaii	MH879469	IC_50_ (cytotoxicity) 0.591–1.855 μM	[47]
Waikikiamide B (**98**)	*Aspergillus* sp. FM242	soil, Waikiki beach of Oahu, Honolulu, Hawaii	MH879469	–	[47]
Waikikiamide C (**99**)	*Aspergillus* sp. FM242	soil, Waikiki beach of Oahu, Honolulu, Hawaii	MH879469	IC_50_ (cytotoxicity) 1.127–1.805 μM	[47]
Aspamides A-D (**100**–**103**)	*A. versicolor* DY180635	Sea crab (*Chiromantes haematocheir*), Zhoushan, Zhejiang, China	MT361076	–	[48]
Aspamides F-G (**104**–**105**)	*A. versicolor* DY180635	Sea crab (*Chiromantes haematocheir*), Zhoushan, Zhejiang, China	MT361076	–	[48]
Sclerotiamide I (**106**)	*A. sclerotiorum* LZDX-33-4	Marine gorgonian, South China Sea	OK012383.1	–	[49]
Sclerotiamide J (**107**)	*A. sclerotiorum* LZDX-33-4	Marine gorgonian, South China Sea	OK012383.1	Inhibited NLRP3 inflammasome activation	[49]
Sclerotiamide K (**108**)	*A. sclerotiorum* LZDX-33-4	Marine gorgonian, South China Sea	OK012383.1	MIC (antimicrobial) 4–16 µM	[49]
Sclerotiamides L–R (**109**–**115**)	*A. sclerotiorum* LZDX-33-4	Marine gorgonian, South China Sea	OK012383.1	–	[49]
11-methylneoechinulin E (**116**)	*Aspergillus* sp. EGF 15-0-3	Soft coral, South China Sea	FJ941865.1	–	[50]
Variecolorin M (**117**)	*Aspergillus* sp. EGF 15-0-3	Soft coral, South China Sea	FJ941865.1	–	[50]
(+)-variecolorin G (**118**)	*Aspergillus* sp. EGF 15-0-3	Soft coral, South China Sea	FJ941865.1	–	[50]
Versicomide E (**119**)	*A. versicolor* AS-212	Deep-sea coral *Hemicorallium* cf. *imperiale*, Magellan Seamounts	OP009765.1	–	[51]
Cottoquinazoline H (**120**)	*A. versicolor* AS-212	Deep-sea coral *Hemicorallium* cf. *imperiale*, Magellan Seamounts	OP009765.1	MIC (antimicrobial) 9.0–18.1 µM	[51]
Versicoxepines A–D (**121**–**124**)	*A. versicolor* AS-212	Deep-sea coral *Hemicorallium* cf. *imperiale*, Magellan Seamounts	OP009765.1	–	[52]
Asperopiperazine A (**125**)	*Aspergillus* sp. DY001	Sea tunicate *Didemnum* sp., Jizan, Saudi Red Sea coast	MN818770	MIC (antimicrobial) 8 µM; IC_50_ (cytotoxicity) 15.1 ± 0.1–24.3 ± 0.2 μM	[53]
Asperopiperazine B (**126**)	*Aspergillus* sp. DY001	Sea tunicate *Didemnum* sp., Jizan, Saudi Red Sea coast	MN818770	MIC (antimicrobial) 4–8 µM; IC_50_ (cytotoxicity) 16.2 ± 0.1–26.3 ± 0.3 μM	[53]
Aspergiamide A (**127**)	*Aspergillus* sp. 16-5c	Leaves of *S. apetala*, a mangrove, Hainan Island, China	JX993829	IC_50_ (α-glucosidase-inhibitory) 18.2 Μm	[54]
Aspergiamide B (**128**)	*Aspergillus* sp. 16-5c	Leaves of *S. apetala*, a mangrove, Hainan Island, China	JX993829	–	[54]
Aspergiamide C (**129**)	*Aspergillus* sp. 16-5c	Leaves of *S. apetala*, a mangrove, Hainan Island, China	JX993829	IC_50_ (α-glucosidase-inhibitory) 83.9 μM	[54]
Aspergiamides D-F (**130**–**132**)	*Aspergillus* sp. 16-5c	Leaves of *S. apetala*, a mangrove, Hainan Island, China	JX993829	–	[54]
24,25-dihydroxyvariecolorin G (**133**)	*A. chevalieri* CS-122	Deep-sea cold-seep sediment, South China Sea	OM304365.1	MIC (anti-*E. coli*) 4 µg/mL	[55]
25-hydroxyrubrumazine B (**134**)	*A. chevalieri* CS-122	Deep-sea cold-seep sediment, South China Sea	OM304365.1	MIC (antibacterial) 16–32 µg/mL	[55]
22-chloro-25-hydroxyrubrumazine B (**135**)	*A. chevalieri* CS-122	Deep-sea cold-seep sediment, South China Sea	OM304365.1	MIC (anti-*V. harveyi*) 8 µg/mL	[55]
25-hydroxyvariecolorin F (**136**)	*A. chevalieri* CS-122	Deep-sea cold-seep sediment, South China Sea	OM304365.1	MIC (antimicrobial) 32 µg/mL	[55]
27-epi-aspechinulin D (**137**)	*A. chevalieri* CS-122	Deep-sea cold-seep sediment, South China Sea	OM304365.1	Potent broad-spectrum antibacterial activity	[55]
Asterripeptides A–C (**138**–**140**)	*A. terreus* LM.5.2	Mangrove tree leaves *Kandelia candelcoast*, Hoa province, Vietnam, South China Sea	MN788658.1	–	[56]
19S,20-epoxy-18-oxotryprostatin A (**141**)	*A. fumigatus* MF071	Marine sediment, Bohai Sea, China	MN700176	–	[57]
20-hydroxy-18-oxotryprostatin A (**142**)	*A. fumigatus* MF071	Marine sediment, Bohai Sea, China	MN700176	–	[57]
Nigerpiperazine A (**143**)	*A. niger* JX-5	Mangrove plant *Ceriops taga*l, Dongzhaigang, Hainan, China	MK234873	IC_50_ (insecticidal activity) 200 μg/mL	[58]
Oxepinamides H–J (**144**–**146**)	*A. puniceus* SCSIO z021	Deep-sea sediment, Okinawa Trough	KX258801	EC_50_ (transcriptional activation on LXRα) 15–16 μM	[59]
Oxepinamide K (**147**)	*A. puniceus* SCSIO z021	Deep-sea sediment, Okinawa Trough	KX258801	–	[59]
Chevalinulins A (**148**)	*A. chevalieri* CS-122	Deep-sea cold seep, South China Sea	OM304365	Proangiogenic activity at the concentrations of 40 μg/mL	[60]
Chevalinulins B (**149**)	*A. chevalieri* CS-122	Deep-sea cold seep, South China Sea	OM304365	Proangiogenic activity at the concentration of 80 μg/mL	[60]
Sclerotiotide M (**150**)	*A. ochraceopetaliformis*	Seawater, Dongshan Island, Fujian, China	MW047023	–	[63]
	DSW-2				
JG002CPA (**151**)	*A. allahabadii* JG002	Underwater sediment, the coast of Jeju-do, Korea	MK424488	–	[64]
JG002CPB (**152**)	*A. allahabadii* JG002	Underwater sediment, the coast of Jeju-do, Korea	MK424488	IC_50_ (antimicrobial) 47.8–104.3 μM	[64]
FJ120DPA (**153**)	*A. ochraceopetaliformis* FJ120	Underwater sediment, the coast of Jeju-do, Korea	KF384187	–	[64]
FJ120DPB (**154**)	*A. ochraceopetaliformis* FJ120	Underwater sediment, the coast of Jeju-do, Korea	KF384187	–	[64]
Aspertides A–C (**155**–**157**)	co-culture of *A. tamarii* MA-21 and *A. insuetus* SD-512	Mangrove plant *S. paracaseolaris*, Wenchang, Hainan, China and deep-sea sediment, South China Sea	HQ891663 and MN696202	–	[65]
Aspertide D (**158**)	co-culture of *A. tamarii* MA-21 and *A. insuetus* SD-512	Mangrove plant *S. paracaseolaris*, Wenchang, Hainan, China and deep-sea sediment, South China Sea	HQ891663 and MN696202	MIC (antibacterial) 8–32 µg/mL	[65]
Aspertide E (**159**)	co-culture of *A. tamarii* MA-21 and *A. insuetus* SD-512	Mangrove plant *S. paracaseolaris*, Wenchang, Hainan, China and deep-sea sediment, South China Sea	HQ891663 and MN696202	MIC (antibacterial) 8–16 µg/mL	[65]
Japonamides A–B (**160**–**161**)	*A. japonicus* MCCC 3A00261	Marine sponge, Arctic 6700-4 sea area	HM573340	–	[66]
Pseudoviridinutans A–E (**162**–**166**)	*A. pseudoviridinutans* TW58-5	Marine sediment, Kueishantao, Taiwan	OQ405296	–	[67]
Pseudoviridinutan F (**167**)	*A. pseudoviridinutans* TW58-5	Marine sediment, Kueishantao, Taiwan	OQ405296	Inhibited LPS and stimulated NO production	[67]
Pseudoviridinutan G (**168**)	*A. pseudoviridinutans* TW58-5	Marine sediment, Kueishantao, Taiwan	OQ405296	–	[67]
Petrosamides A-C (**169**–**171**)	*Aspergillus* sp. 151304	Marine sponge *Petrosia* sp., Yongxing Island, China	SUB7318612	IC_50_ (PL-inhibitory activities) 0.5 ± 0.1–7.6 ± 1.5 μM	[68]
Cotteslosin C (**172**)	co-culture of *A. versicolor* 8.1.3a and *B. subtilis* 168 trpC2	Sponge and laboratory strain	KY174984	–	[69]
Asperhiratide (**173**)	*A. hiratsukae* SCSIO 5Bn1003	Soft coral, South China Sea	KY806121.1	–	[70]
Versicolide A (**174**)	*A. versicolor* PS108-62	Marine sediment, Arctic Ocean	OP807024	–	[71]
Maribasin C (**175**)	*Aspergillus* sp. SCSIO 41501	Marine gorgonian *Melitodes squamata* Nutting, the South China Sea, Sanya, Hainan	JN851015	MIC (antifungal) 6.25–50 µg/disc	[21]
Maribasin D (**176**)	*Aspergillus* sp. SCSIO 41501	Marine gorgonian *Melitodes squamata* Nutting, the South China Sea, Sanya, Hainan	JN851015	MIC (antifungal) 3.12–25 µg/disc	[21]
Maribasin E (**177**)	*Aspergillus* sp. SCSIO 41501	Marine gorgonian *Melitodes squamata* Nutting, the South China Sea, Sanya, Hainan	JN851015	MIC (antifungal) 12.5–50 µg/disc	[21]
Sclerotiotide M (**178**)	*A. insulicola* HDN151418	Sponge, Prydz Bay, Antarctica	MT898544	MIC (antimicrobial) 1.56–12.5 µM	[72]
Sclerotiotide N (**179**)	*A. insulicola* HDN151418	Sponge, Prydz Bay, Antarctica	MT898544	MIC (antimicrobial) 1.56–25.0 µM	[72]
Sclerotiotide O (**180**)	*A. insulicola* HDN151418	Sponge, Prydz Bay, Antarctica	MT898544	–	[72]
Caletasin (**181**)	*Aspergillus* sp. MEXU 27854	Mairne sand, Caleta Bay, Mexico	KY406733	–	[73]
Chaetominine A (**182**)	*A. fumigatus* MF029	Marine sponge *Hymeniacidon perleve*, Bohai Sea, China	MH974808	–	[75]
Aspertoryadins H-J (**183**–**185**)	*Aspergillus* sp. HNMF114	*Sanguinolaria chinensi*, Haikou Bay	MK732953	–	[76]
Chaetominines A (**186**) and B (**187**)	*A. versicolour * SCSIO XWS04 F52	Marine sponge *Callyspongia* sp., Xuwen County, Guangdong Province, China	MN788648	IC_50_ (cytotoxicity) 7.5–24.5 µM	[77]
Puniceloid E (**188**)	*A. puniceus* FAHY0085	Marine coral, South China Sea	OQ825098	–	[36]
Puniceloid F (**189**)	*A. puniceus* FAHY0085	Marine coral, South China Sea	OQ825098	EC_50_ (transcriptional activation on LXRα) 2 µM	[36]
Puniceloid G (**190**)	*A. puniceus* FAHY0085	Marine coral, South China Sea	OQ825098	–	[36]
2-(4-hydroxybenzyl)-4-(3-acetyl)	*A. sydowii* SW9	Seawater, Yangma Island, Yantai, China	MN696205	MIC (antibacterial) 8–16 µg/mL	[78]
quinazolin-one (**191**)					
Aspertoryadins A–E (**192**–**196**)	*Aspergillus* sp. HNMF114	*Sanguinolaria chinensi*, Haikou Bay	MK732953	–	[79]
Aspertoryadin F (**197**)	*Aspergillus* sp. HNMF114	*Sanguinolaria chinensi*, Haikou Bay	MK732953	MIC (antifungal) 32 µg/well	[79]
Aspertoryadin G (**198**)	*Aspergillus* sp. HNMF114	*Sanguinolaria chinensi*, Haikou Bay	MK732953	MIC (antifungal) 32 µg/well	[79]
2-*epi*-tryptoquivaline F (**199**)	*A. fumigatus* H22	Seawater, Western Pacific	NRRL 163 s	–	[17]
Protuboxepin K (**200**)	*Aspergillus* sp. BFM-0085	Sediment, Tokyo Bay, Tokyo, Japan	–	IC_50_ (cytotoxicity) 4.7 µM	[80]
Felicarnezolines A–B (**201**–**202**)	co-culture of *Amphichorda* sp. KMM 4639 and *A. carneus* KMM 4638	Van Phong Bay, the South China Sea, Vietnam, and Brown alga *Laminaria sachalinensis*, Kunashir Island	OQ344667	–	[81]
Felicarnezoline C (**203**)	co-culture of *Amphichorda* sp. KMM 4639 and *A. carneus* KMM 4638	Van Phong Bay, the South China Sea, Vietnam, and Brown alga *Laminaria sachalinensis*, Kunashir Island	OQ344667	IC_50_ (cytotoxicity) 92.5 ± 3.1 µM	[81]
Felicarnezoline D (**204**)	co-culture of *Amphichorda* sp. KMM 4639 and *A. carneus* KMM 4638	Van Phong Bay, the South China Sea, Vietnam, and Brown alga *Laminaria sachalinensis*, Kunashir Island	OQ344667	IC_50_ (cytotoxicity) 68.7 ± 1.6–72.9 ± 2.8 µM	[81]
Felicarnezoline E (**205**)	co-culture of *Amphichorda* sp. KMM 4639 and *A. carneus* KMM 4638	Van Phong Bay, the South China Sea, Vietnam, and Brown alga *Laminaria sachalinensis*, Kunashir Island	OQ344667	IC_50_ (cytotoxicity) 83.8 ± 5.5–86.3 ± 2.3 µM	[81]
(-)-isoversicomide A (**206**)	*A. versicolor* PS108-62	Marine sediment, Arctic Ocean	OP807024	–	[71]
29-hydroxyfumiquinazoline C (**207**)	*A. fumigatus* SD-406	Deep-sea sediment, the East China Sea	MT635279	–	[20]
(±)-17-hydroxybrevianamide N (**208**)	*Aspergillus* sp. CHNSCLM-0151	Soft coral, South China Sea	KY235298	–	[82]
(±)-N1-methyl-17-hydroxybrevianamide N (**209**)	*Aspergillus* sp. CHNSCLM-0151	Soft coral, South China Sea	KY235298	–	[82]
Puniceloids A–D (**210**–**213**)	*A. puniceus* SCSIO z021	Deep-sea sediment, Okinawa Trough	KX258801	EC_50_ (transcriptional activation on LXRα) 1.7–5.3 µM	[59]
Novobenzomalvin D (**214**)	*A. terreus* SCAU011	Mangrove plant *Rhizophora* *stylosa*, Techeng Isle, China	KY827341	COX-2 inhibition rate of 91.1% at 20 nM	[83]
Tryptoquivaline Y (**215**)	*A. felis* FM324	Hawaiian beach soil	MZ227547	–	[84]
2-methyl-versiquinazoline C (**216**)	*A. flavipes* PJ03-11	Wetland mud, Panjin Red Beach National Nature Reserve, Liaoning Province, China	KT809365	–	[85]
Fumigatosides G-H (**217**–**218**)	*A. fumigatus* SAl12	Leaves of mangrove plant Sonneratia apetala Buch.-Ham., Dongzhaigang National Nature Reserve, south China’s Hainan Province	–	–	[86]
Puniceusine O (**219**)	*A. puniceus* SCSIO z021	Deep-sea sediment, Okinawa Trough	KX258801	–	[88]
(±)-puniceusine P (**220**)	*A. puniceus* SCSIO z021	Deep-sea sediment, Okinawa Trough	KX258801	–	[88]
Puniceusines A-B (**221**–**222**)	*A. puniceus* SCSIO z021	Deep-sea sediment, Okinawa Trough	KX258801	–	[89]
Puniceusine C (**223**)	*A. puniceus* SCSIO z021	Deep-sea sediment, Okinawa Trough	KX258801	IC_50_ (CD45-inhibitory activities) 8.4 µM	[89]
Puniceusine D (**224**)	*A. puniceus* SCSIO z021	Deep-sea sediment, Okinawa Trough	KX258801	IC_50_ (CD45-inhibitory activities) 5.6 μM; IC_50_ (cytotoxicity) 11.0 µM; MIC (anti-*E. coli*) 100 µg/mL	[89]
Puniceusines E-M (**225**–**233**)	*A. puniceus* SCSIO z021	Deep-sea sediment, Okinawa Trough	KX258801	–	[89]
Puniceusine N (**234**)	*A. puniceus* SCSIO z021	Deep-sea sediment, Okinawa Trough	KX258801	MIC (antibacterial) 100 µg/mL	[89]
2-(quinoline-8-carboxamido)	*Aspergillus* sp. SCSIO06786	Deep-sea sediment, Indian Ocean	MN203718	–	[90]
benzoic acid (**235**)					
perinadine B-C (**236**–**237**)	*Aspergillus* sp. LS116	Marine sponge *Haliclona* sp., Lingshui, Hainan, China	FJ864703	MIC (antibacterial) 32–64 µg/mL	[92]
Ochraceopetalin (**238**)	*A. ochraceopetaliformis* FJ120	Marine sediment, Jeju-do, Korea	KF384187	IC_50_ (cytotoxicity) 6.8–9.5 µM	[93]
Asperorydines N-P (**239**–**241**)	*A. flavus* SCSIO F025	Deep-sea sediment, the central South China Sea	KF682370	–	[94]
Variotin B (**242**)	*A. unguis* IV17-109	Deep-sea shrimp, Indian Ocean	OL700797	IC_50_ (anti-inflammatory) 20.0 µM	[95]
(*E*)-6-hydroxy-5-(1-propenyl)-1,2-dihydropyrano [3,2-b]pyrrole-3,7-dione (**243**)	*Aspergillus* sp. DM94	Rhizosphere soil of mangrove *Bruguiera gymnorrhiza* (L.) Poir	M20191003	–	[96]
Cephalimysins M–N (**244**–**245**)	*A. fumigatus* CUGBMF170049	Marine sediment, Bohai Sea, China	MK453215	–	[97]
Azaspirenes A–E (**246**–**250**)	*A. micronesiensis* NF666	Marine mud, South China Sea	–	–	[98]
10R-15-methylpseurotin A (**251**)	*A. fumigatus* SD-406	Deep-sea sediment, the East China Sea	MT635279	MIC (antifungal) 16 µM	[20]
Pseurotin I (**252**)	*A. felis* FM324	Hawaiian beach soil	MZ227547	IC_50_ (NF-κB-inhibitory activities) 30.9 µM	[84]
(±)-asperazepanone A (**253**)	*A. candidus* CHNSCLM-0393	Nansha Islands coral reef, South China	MF681708	–	[99]
(+)-asperazepanone B (**254**)	*A. candidus* CHNSCLM-0393	Nansha Islands coral reef, South China	MF681708	Against nitric oxide (NO) production with an inhibition rate of 43 ± 4% at the concentration of 1 μM	[99]
Asperorydine Q (**255**)	*A. flavus* GXIMD 02503	Coral *Porites lutea*, Guangxi Zhuang Autonomous Region, China	MT510157 and MT510158	IC_50_ (NF-κB-inhibitory activities) 14.1 ± 1.5 µM	[100]
pyranonigrin L (**256**)	*A. fumigatus* SAS10	Mangrove, Dongzhai Harbor, Hainan Province, China	–	–	[101]
Asperalins A–B (**257**–**258**)	*A. alabamensis* SYSU-6778	Seagrass *E. acoroides*, Dongzhai Port, Hainan Island, China	ON845600	–	[102]
Asperalins C–D (**259**–**260**)	*A. alabamensis* SYSU-6778	Seagrass *E. acoroides*, Dongzhai Port, Hainan Island, China	ON845600	MIC (antimicrobial) 5–10.1 µM	[102]
Asperalin E (**261**)	*A. alabamensis* SYSU-6778	Seagrass *E. acoroides*, Dongzhai Port, Hainan Island, China	ON845600	MIC (antimicrobial) 2.2 µM	[102]
Asperalin F (**262**)	*A. alabamensis* SYSU-6778	Seagrass *E. acoroides*, Dongzhai Port, Hainan Island, China	ON845600	MIC (antimicrobial) 10.9–87.3 µM	[102]
22-epi-aflaquinolone B (**263**)	co-culture of *A. versicolor* 8.1.3a and *B. subtilis* 168 trpC2	Sponge and laboratory strain	KY174984	–	[69]
Aspergillspins C–E (**264**–**266**)	*Aspergillus* sp. SCSIO 41501	Marine gorgonian *Melitodes squamata* Nutting, the South China Sea, Sanya, Hainan	JN851015	–	[23]
Citriquinolinones A–B (**267**–**268**)	*A. versicolor* 170217	Deep-sea whale *Mesoplodon densirostris*, Ningde, East China Sea	SUB13826338	–	[103]
Circumdatin M (**269**)	*Aspergillus* sp. FM242	Soil, Waikiki beach of Oahu, Honolulu, Hawaii	MH879469	–	[104]
Kipukasins M (**270**) and N (**271**)	*A. versicolor* TJ-LHQ-AV507	Sea mud, South China Sea	2081031	–	[105]
Asperpteridinate A (**272**)	*A. austroafricanus* Y32-2	Seawater, Indian Ocean	MK267449	–	[13]
Pyripyropene U (**273**)	*Aspergillus* sp. SCSIO41420	Marine sponge, Weizhou Island, Guangxi, China	NR_ OP363213	–	[106]
Aspernigrin E (**274**)	*A. fumigatus* SAS10	Mangrove, Dongzhai Harbor, Hainan Province, China	–	–	[101]
(*S*)-3-hydroxy-2,7-dimethylfuro [3,4-b]pyridin-5(7*H*)-one (**275**)	*Aspergillus* sp. SCSIO41405	Coral, Luhuitou waters, Sanya Bay, South China Sea	–	–	[107]
Asperalumazine A (**276**)	*A. alabamensis* SYSU-6778	Seagrass *E. acoroides*, Dongzhai Port, Hainan Island, China	ON845600	–	[102]
Fiscpropionate D (**277**)	*A. fischeri* FS452	Deep-sea sludge, Indian Ocean	KF294264	IC_50_ (*M*ptpB-inhibitory activities) 11 μM	[108]
Fiscpropionate E (**278**)	*A. fischeri* FS452	Deep-sea sludge, Indian Ocean	KF294264	–	[108]
Rhizoaspergillin A (**279**)	*Aspergillus* sp. A1E3	Mangrove *Rhizophora mucronata*, Trang Province	–	–	[109]
Acremolin D (**280**)	*A. sydowii* MCCC 3A00324	Deep-sea sediment, South Atlantic Ocean	MN918102	Inhibition rate (cytotoxicity) of 25.1–30.6% at the concentration of 20 µM	[110]
Phomaligol H (**281**)	*A. flavus* BB1	Marine shellfish *Meretrix meretrix*, Hailing Island, Yangjiang, China	MT584825	IC_50_ (cytotoxicity) 65.53 µM	[111]
Pyrasplorines A–C (**282**–**284**)	*A. verisicolor* HDN11-84	*Thespesia populnea*, Guangxi Province, China	KU950433	–	[112]
Deg-pyrasplorine B (**285**)	*A. verisicolor* HDN11-84	*Thespesia populnea*, Guangxi Province, China	KU950433	–	[112]
Versicoloid A (**286**)	*A. verisicolor* HDN11-84	*Thespesia populnea*, Guangxi Province, China	KU950433	–	[112]
Penilumamide K (**287**)	Aspergillus sp. SCSIO 41029	Deep-sea sediment, South China Sea	MH591418.1	IC_50_ (α-glucosidase inhibitory) 18.61 μM	[113]
(6-benzyl-1-isopentyl-4-oxo-1,4-dihydropyridin-3-yl)-carboxamide (**288**)	*Aspergillus* sp. DM94	Rhizosphere soil of mangrove *Bruguiera gymnorrhiza* (L.) Poir	M20191003	–	[96]
Flavipesides A–C (**289**–**291**)	*A. flavipes* 164013	Cyanobacterium *Lyngbya majuscula*, South China Sea	–	IC_50_ (PL inhibitory activities) 0.07 ± 0.01–0.23 ± 0.03 μM	[114]
kipukasins K–L (**292**–**293**)	*A. versicolor* XS-20090066	Gorgonian *Dichotella gemmacea*, Xisha Islands coral reef, South China Sea	MN880095	–	[115]
